# Role and mechanism of IGFBP5 in the real-ambient particulate matter exposure-induced chronic lung injury

**DOI:** 10.3389/fphar.2025.1604301

**Published:** 2025-06-25

**Authors:** Ningning Chen, Yuan Qi, Wanli Ma, Xiaoxiao Zhu, Xiaoying Li

**Affiliations:** ^1^ Department of Neonatology, Children’s Hospital Affiliated to Shandong University (Jinan Children’s Hospital), Jinan, China; ^2^ School of Basic Medical Sciences, Shandong University, Jinan, China; ^3^ School of Public Health, Qingdao University, Qingdao, China

**Keywords:** PM, IGFBP5, MiR-33a-5p, oxidative damage, lung injury

## Abstract

**Background:**

Inflammation and oxidative stress are the main pathological processes of particulate matter (PM)-induced lung injury. Insulin-like growth factor binding protein 5 (IGFBP5) is an important secretory protein related to inflammation and oxidative damage in several tissues, whereas its roles in PM-induced lung adverse effects remain largely unexplored.

**Methods:**

In the present study, mice were housed in an individual ventilated cage (IVC)-based real-ambient PM exposure system for eight weeks. Transcriptomics was employed to analyze gene expression alterations.

**Results:**

IGFBP5 was significantly downregulated after PM exposure. Functional investigations demonstrated that IGFBP5 downregulation exacerbated PM-induced oxidative damage, as evidenced by elevated levels of reactive oxygen species (ROS) and malondialdehyde, as well as decreased levels of superoxide dismutase 2 (SOD2). Conversely, IGFBP5 overexpression effectively rescued these oxidative stress phenotypes. Mechanistically, IGFBP5 downregulation attenuated extracellular signal-regulated kinase 1/2 (ERK1/2) phosphorylation, thereby impairing SOD2 catalytic activity and amplifying ROS accumulation. Co-treatment with si-IGFBP5 and ERK1/2 signaling pathway inhibitor PD98059 could further aggravate the production of ROS in cells. Moreover, microRNAs (miRNAs) are an important class of gene expression regulators. We found that the upregulated hsa-miR-33a-5p repressed IGFBP5 translation by forming a silencing complex with Argonaute protein 2 (AGO2) in a real-ambient PM exposure system, which further led to the suppression of the ERK1/2-SOD2 signaling pathway and increased levels of ROS.

**Conclusion:**

This study revealed that the downregulation of IGFBP5 promoted oxidative damage in lung cells by inhibiting the IGFBP5-ERK1/2-SOD2 pathway, and targeted inhibition of hsa-miR-33a could alleviate PM-induced lung injury by upregulating IGFBP5.

## 1 Introduction

Air pollution has emerged as an urgent environmental and public health issue worldwide (Fuller et al., 2022; Ran et al., 2025). As the primary air pollutant, atmospheric particulate matter (PM) has always received widespread attention due to its small particle size, large surface area, and strong ability to absorb toxins (Vaccarella et al., 2025). It is well known that the lungs are one of the organs most susceptible to the effects of ambient PM (Hou et al., 2024). A substantial body of epidemiological evidence indicates that there is a strong relationship between ambient PM exposure and human lung disorders. For example, prolonged exposure to PM is associated with the exacerbation of chronic obstructive pulmonary disease (COPD) and even lung cancer (Goobie et al., 2024; Hu et al., 2025; Jiang et al., 2024). Unfortunately, the mechanisms of lung toxicity caused by PM exposure remain poorly understood and require further investigation.

Insulin-like growth factor binding protein 5 (IGFBP5) is the most conserved member of the IGFBP family. A salient feature of this family is that its members competitively bind to IGF with IGF receptor (IGFR), thereby augmenting or inhibiting IGF actions under different conditions (Bach, 2018). Intriguingly, it has been found in recent years that IGFBP5 exhibits diverse biological functions independent of IGF. For instance, the inflammatory response, oxidative damage and cell migration induced by IGFBP5 are primarily mediated through the ERK1/2 or p38 MAPK pathways (Allard and Duan, 2018; Song et al., 2022; Yasuoka et al., 2019), which greatly extends the function of IGFBP5 in diseases. Despite this, most studies are limited to the role of IGFBP5 in cancer, including breast cancer (Dittmer, 2022), colorectal cancer (Deng et al., 2022), and ovarian cancer (Fan et al., 2024). Recent evidence shows that 2,3,7,8-Tetrachlorodibenzo-p-dioxin (TCDD), a typical organic pollutant, exerts toxic effects on human breast carcinoma cells and endometrial carcinoma cells by activating IGFBP5 (Tanaka et al., 2007), indicating that IGFBP5 may act as an endogenous regulatory factor involved in tissue damage induced by environmental factors. However, the role of IGFBP5 in PM-induced lung injury is still unclear.

MicroRNAs (miRNAs) are a class of ∼22 nt non-coding single-stranded RNA. As an essential epigenetic factor, miRNAs have the capacity to regulate gene expression at the post-transcriptional level, and this regulatory function allows miRNAs to have a wide range of biological functions (Gebert and MacRae, 2019). In parallel, miRNAs are especially attractive in a biomarker setting because of their stability in human blood and other body fluids, as well as their disease-specific expression patterns. To date, a significant number of dysregulated miRNAs have been identified in response to exposure to PM (Chen et al., 2024; Hou et al., 2018; Wang et al., 2020). However, it is still unclear whether dysregulated miRNAs after PM exposure participate in the regulation of IGFBP5 expression.

In this study, we first analyzed alterations in mRNAs expression profiles in the lungs of mice housed in a real-ambient PM exposure system. We found that the expression of IGFBP5 was significantly downregulated in lung tissues of mice exposed to PM. Further investigations confirmed that IGFBP5 was primarily influenced by the organic extract fractions of PM rather than the water-soluble fractions. Functionally, our data showed that inhibition of IGFBP5 *in vitro* significantly increased PM-induced ROS production in lung cell lines A549 and BEAS-2B, and the mechanism involved was found to be associated with IGFBP5-mediated non-canonical MAPK (ERK1/2) signaling pathway. Moreover, exposure to real-ambient PM resulted in altered miRNA expression profiles in the lungs of mice. The upregulated miR-33a-5p, as an upstream regulator of IGFBP5, could directly interact with the transcript by targeting its 3′UTR, leading to the repression of IGFBP5 translation. These findings will facilitate a deeper understanding of the potential mechanisms of lung injury caused by real-ambient PM exposure.

## 2 Materials and methods

### 2.1 Animal models

The individual ventilation cage (IVC)-based real-ambient PM exposure system was placed in Shijiazhuang (a city with the highest PM concentration in China). The method of PM exposure has been reported in our previous study (Li et al., 2019). Briefly, male C57/BL6J mice (6 weeks) were purchased from the Model Animal Research Center of Nanjing University (Nanjing, Jiangsu, China) and were maintained under a 12-h light-dark cycle with free access to water and food. After 1-week adaptation, mice were randomly divided into two groups: the control group and PM exposure group (n = 10). The control group of mice was housed in an air filtration (AF) control chamber, where three high-efficiency particulate air (HEPA) filters were placed to remove all particulate matter. The other was placed in a PM exposure chamber, where the mice inhaled PM concentrations equal to those in the outdoor atmosphere. Animals were exposed to unfiltered or filtered air for 16 h per day and 7 days per week. After 8 weeks, all the mice were euthanized and subjected to the following experiments. All experimental protocols were approved by the Institutional Animal Care and Use Committee of Qingdao University (QDU-AEC-2022044), and carried out under the institutional guidelines for ethical animal use.

### 2.2 Cell culture and treatment

Human lung cancer cell line A549, human normal lung epithelial cells (BEAS-2B), and embryo kidney cell line HEK293T (293T) were purchased from American Tissue Type Culture Collection (ATCC, Manassas, VA). All cells were cultured in DMEM medium (BasalMedia Technologies, Shanghai, China) containing 10% fetal bovine serum (VivaCell, Shanghai, China) and maintained in a humidified incubator containing 5% CO_2_ at 37°C.

The collection and component extraction of PM were performed according to previous studies (Li et al., 2019). Briefly, the PM was collected using polytetrafluoroethylene filters at a flow rate of 1.05 L/min, 24 h per day for 8 weeks. The collected sampling membranes were cut into small pieces of about 1 cm × 1 cm and placed in a conical flask. After adding ultrapure water, PM particles were obtained through sonication, shaking, elution, filtration, and freeze-drying. For PM exposure studies, cells were initially seeded in 12-well plates, cultured overnight, and treated with 0, 50, and 100 μg/mL PM. The dose selection was based on our previous findings that a dose of 50 μg/mL had low cytotoxicity (less than 20%), while 100 μg/mL showed significant cytotoxicity with a 31% inhibition of cell viability. For cells transfection, miRNA mimics, miRNA inhibitors, small interfering RNAs (siRNAs) targeting IGFBP5 and AGO2, IGFBP5 overexpression vector (Flag-IGFBP5) and their corresponding negative controls were purchased from GenePharma (Shanghai, China), and then transiently transfected into cells using Lipofectamine RNAiMAX (Invitrogen, Carlsbad, CA, USA). The transfected cells were incubated for another 48 h, and harvested for further experiments.

### 2.3 RNA extraction and quantification

Total RNAs were isolated using Trizol reagent (Invitrogen) in accordance with the manufacturer’s instructions. cDNA was then synthesized using the RT reagent Kit (Takara, Dalian, China) for quantitative real-time PCR (qRT-PCR) assays of mRNA or the Revert Aid First Strand cDNA Synthesis Assay Kit (ThermoFisher Scientific, Waltham, MA, USA) for the miRNA qRT-PCR assays. qRT-PCR was performed using QuantiNova™ SYBR Green PCR reagent (Qiagen, Duesseldorf, Germany) on the LightCycler^®^ 480 System (Roche, Basel, Switzerland). ACTB mRNA and U6 were used as internal loading controls for mRNA and miRNA, respectively. All primers used in this study were listed in Supplementary Table S1. Each sample was analyzed in triplicate, and analysis was carried out using the 2^−ΔΔCT^ method.

### 2.4 Western blot assay

Total proteins in cells were extracted using RIPA (Beyotime, Shanghai, China) containing 1 mM PMSF and were quantified with BCA reagent (Thermo Fisher Scientific). Equal amounts of protein were then separated by sodium dodecyl sulfate-polyacrylamide gel electrophoresis (SDS-PAGE), transferred onto a polyvinylidene fluoride (PVDF) membrane, blocked in 5% skim milk, and incubated with primary antibodies at 4 °C. After HRP-conjugated secondary antibody incubation, the target band signals were detected using the ECL Western blotting substrate (GE). All antibodies used in this study are listed in Supplementary Table S2.

### 2.5 Biochemical analysis

The ROS concentration was measured using a Reactive Oxygen Species Assay Kit (Beyotime) according to the manufacturer’s instructions. The SOD and MDA levels in the same samples were measured using the corresponding commercial kits (Beyotime) according to the manufacturer’s instructions.

### 2.6 RNA-sequencing analysis

Total RNA was isolated from tissues and cells using Trizol Reagent (Invitrogen). The RNA integrity was assessed by NanoDrop ND-2000 (Thermo), and only the sample with RNA integrity number >7.0 was used for next-generation sequencing. Illumina HiSeq X Ten (Illumina, San Diego, CA, USA) sequencing platform was used to harvested raw data of mRNA, and the Illumina HiSeq 2500 was used for miRNA. After quality control, the threshold of fold-change >2 and *p*-value <0.05 was used to identify the differentially expressed mRNAs and miRNAs.

### 2.7 In silico analysis

The Kyoto Encyclopedia of Genes and Genomes (KEGG) (http://www.genome.jp/kegg/) was used to analyze the functions of differentially expressed mRNAs. Additionally, the binding free energy between miRNA and mRNA was calculated using RNAhybrid tool (https://bibiserv.cebitec.uni-bielefeld.de/rnahybrid/).

### 2.8 Fluorescent-based RNA electrophoretic mobility shift assay (FREMSA)

The miR-33a-5p oligonucleotide was labeled with IRDye™ 800 at its 5′ end, while the IGFBP5 mRNA oligonucleotides, corresponding to the miR-33a-5p response element, were synthesized with or without 5′-modification using Cy5.5™. Detailed steps of the FREMSA assays were as described previously with minor modifications. In brief, the synthesized miRNA and/or mRNA oligonucleotide were incubated in a mixed reaction system (Thermo Scientific) to form the miRNA/mRNA duplex. The mixture was then separated by 6% native PAGE electrophoresis at 4°C in the dark. Finally, fluorescence signals were visualized using the Odyssey CLx Infrared Imaging System (LI-COR Biosciences, Lincoln, NE).

### 2.9 Dual-luciferase reporter gene analysis

To determine the regulatory effect of miR-33a-5p on the activity of its target, the 3′UTR of IGFBP5, which contains the response elements of miR-33a-5p, was subcloned into the MCS region of the PGL3-promoter vector. After co-transfection with recombinant plasmids and miR-33a-5p mimics or inhibitor for 48 h, the luciferase activity in cells was tested using the dual-luciferase kit (Promega, Madison, WI). A pRL-SV40 plasmid expressing Renilla luciferase was used to normalize the Firefly luciferase activity.

### 2.10 Histopathology analysis

For tissue, hematoxylin and eosin (H&E) staining were performed using formalin-fixed and paraffin-embedded lung sections (10 µm) according to the manufacturer’s instructions. Images for H&E in tissue were captured by using CaseViewer 2.0 (Budapest, Hungary) at ×20 magnification.

### 2.11 Statistical analysis

All experiments in this study were repeated more than three times. The data obtained from experiments were presented as the mean ± standard deviation (SD) and analyzed with Student’s t-tests or one-way ANOVA using GraphPad Prism version 9.0 (GraphPad). *P* < 0.05 was considered statistically significant.

## 3 Results

### 3.1 IGFBP5 is downregulated in lung tissue of PM-exposed mice

**FIGURE 1:**
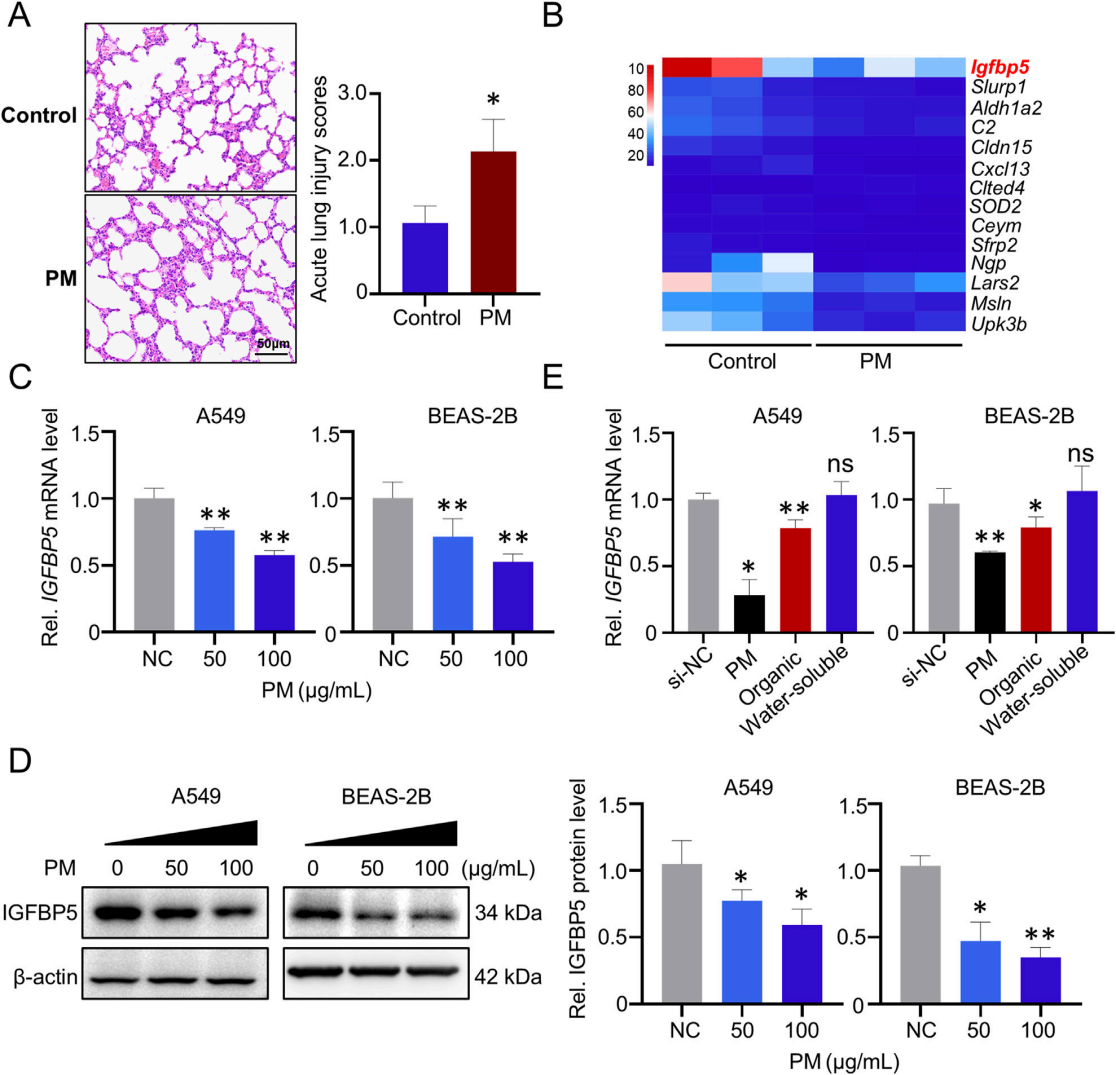
Deregulated genes in lung tissue exposed to PM. **(A)** Representative H&E staining of lung tissues. **(B)** Heatmap of 14 deregulated (fold change >2) genes identified from the lungs of mice exposed to PM (n = 3). **(C)** Validation of the IGFBP5 mRNA level under PM exposure via qRT-PCR. **(D)** The impact of PM on the level of IGFBP5 protein was determined through Western blotting (n = 3). **(E)** The effect of PM organic and water-soluble components on the expression of IGFBP5. Data are presented as mean ± SD (n = 3), *P < 0.05 and **P < 0.01 as indicated.

In the real-ambient PM exposure system, inflammatory infiltration was observed in the lung tissue of mice (Figure 1A). Biochemical analysis showed that oxidative stress levels in the lung tissue of mice exposed to PM were also increased compared to the control group, as evidenced by increased ROS levels and decreased SOD2 levels (Supplementary Figure S1A, B). To further elucidate the effects of real-ambient PM exposure on lung tissue, we then performed transcriptome sequencing on lung tissue samples from C57BL/6J mice housed in a real-ambient PM exposure system. As illustrated in Figure 1B, fourteen RNAs with fold-changes greater than 2 (13 were downregulated and 1 was upregulated) were identified in the PM-exposed mice group, compared to the control group. Among the dysregulated genes, IGFBP5 was most significantly downregulated (downregulated by 3.64-fold, *p* < 0.05). Previous studies have shown that IGFBP5 is involved in the development of lung diseases such as COPD and pulmonary fibrosis by regulating cellular inflammation and oxidative stress, which are also key pathological features of PM-induced lung injury (Li et al., 2021; Yasuoka et al., 2009). Our transcriptomic result prompted us to select IGFBP5 as a candidate gene for further exploration. We then validated the expression of IGFBP5 in lung tissues of mice (Supplementary Figure S1C). Additionally, we found that exposure to PM considerably decreased the expression level of IGFBP5 mRNA in A549 and BEAS-2B cells in a dose-dependent manner (A549: decreased by 23.8% and 42.5% at 50 μg/mL and 100 μg/mL, respectively; BEAS-2B: decreased by 28.9% and 47.7% at 50 μg/mL and 100 μg/mL, respectively) (Figure 1C). Consistently, a diminution in IGFBP5 protein level was also observed in cells following PM exposure (Figure 1D). PM is a complex and heterogeneous mixture of organic and inorganic compounds. To investigate which component in PM is responsible for the downregulation of IGFBP5 expression, we isolated the organic components and water-soluble fraction of PM, and then evaluated their effects on IGFBP5 level, respectively. Interestingly, we found that IGFBP5 was significantly inhibited by the organic extract fractions of PM, but not by the water-soluble fractions (A549: decrease by 21.7%; BEAS-2B: decrease by 20.8%) (Figure 1E).

**FIGURE 2:**
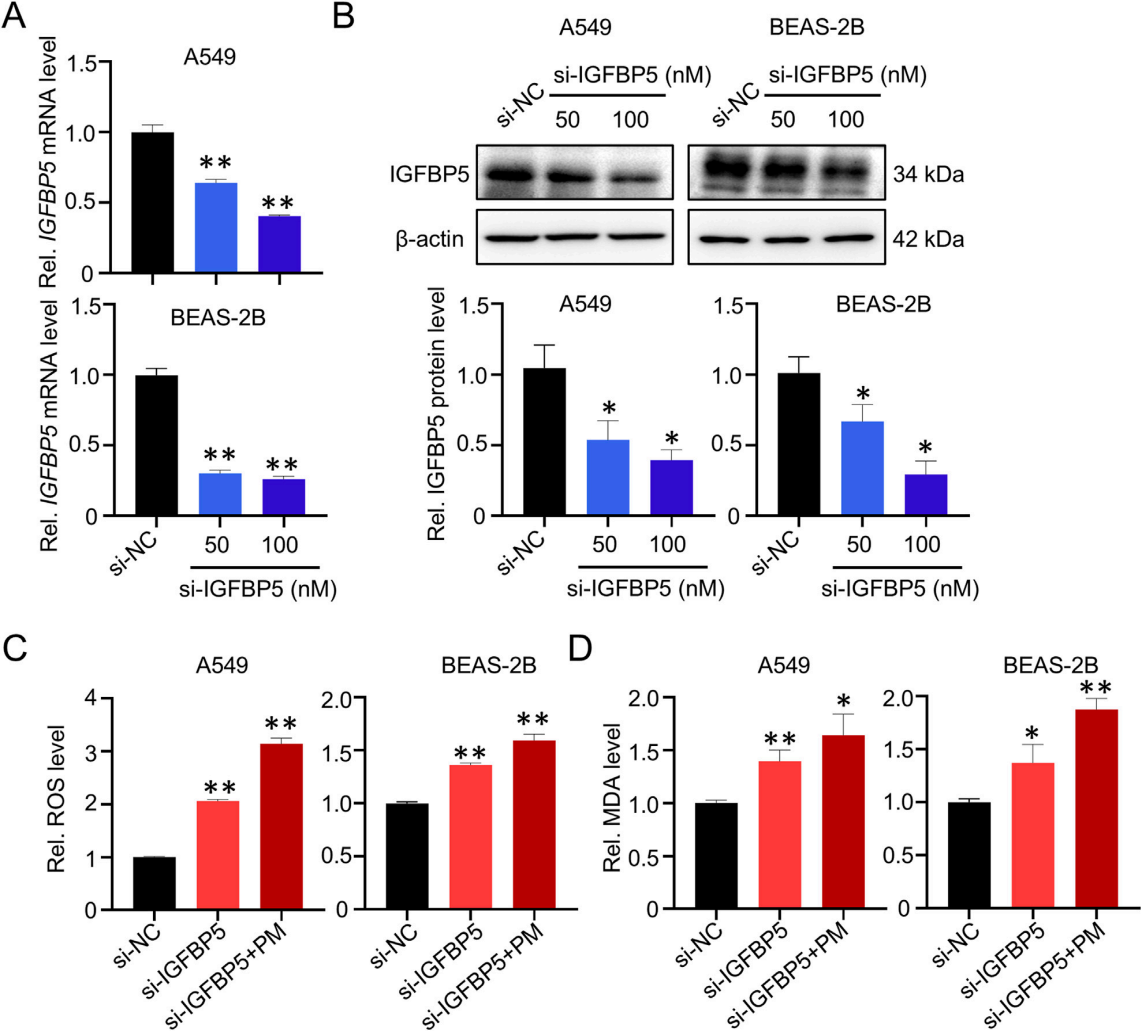
IGFBP5 is related to PM-induced oxidative damage. **(A, B)** Verification of the efficiency of siRNA via qRT-PCR **(A)** and Western blotting **(B)**. **(C, D)** Measurement of intracellular ROS **(C)** and MDA **(D)** levels. A549 and BEAS-2B cells were transfected with si-IGFBP5 for 24 h. The transfected cells were then exposed to 100 μg/mL PM for another 24 h. Data are presented as mean ± SD (n = 3), *P < 0.05 and **P < 0.01 as indicated.

To further investigate the role of IGFBP5 in PM-induced lung injury, we employed siRNA to silence IGFBP5 expression. As depicted in Figures 2A,B, there was a notable reduction in IGFBP5 mRNA and protein levels in cells transfected with siRNA sequences targeting IGFBP5 (si-IGFBP5), compared with cells transfected with negative control sequences (si-NC). In parallel, we detected that both A549 and BEAS-2B cells experienced an increase in intracellular ROS levels (A549: 2.06-fold; BEAS-2B: 1.36-fold) and MDA levels (A549: 1.45-fold; BEAS-2B: 1.42-fold) after treatment with 100 nM si-IGFBP5, compare to the si-NC group (Figures 2C,D). Nonetheless, the levels of inflammatory factors, such as TNFα, IL-1β and IL-6, demonstrated only minor fluctuations in si-IGFBP5 treated lung cells (Supplementary Figure S2). Moreover, we also observed that compared with si-NC, the incorporation of PM into si-IGFBP5 could further induce the production of ROS (A549: 3.14-fold; BEAS-2B: 1.59-fold) and MDA (A549: 1.64-fold; BEAS-2B: 1.87-fold) (Figures 2C,D). These data suggest that oxidative damage caused by IGFBP5 downregulation may be the pivot for ambient PM exposure-induced lung cell injury.

### 3.2 IGFBP5 silencing inhibits SOD2 expression in lung cells

**FIGURE 3:**
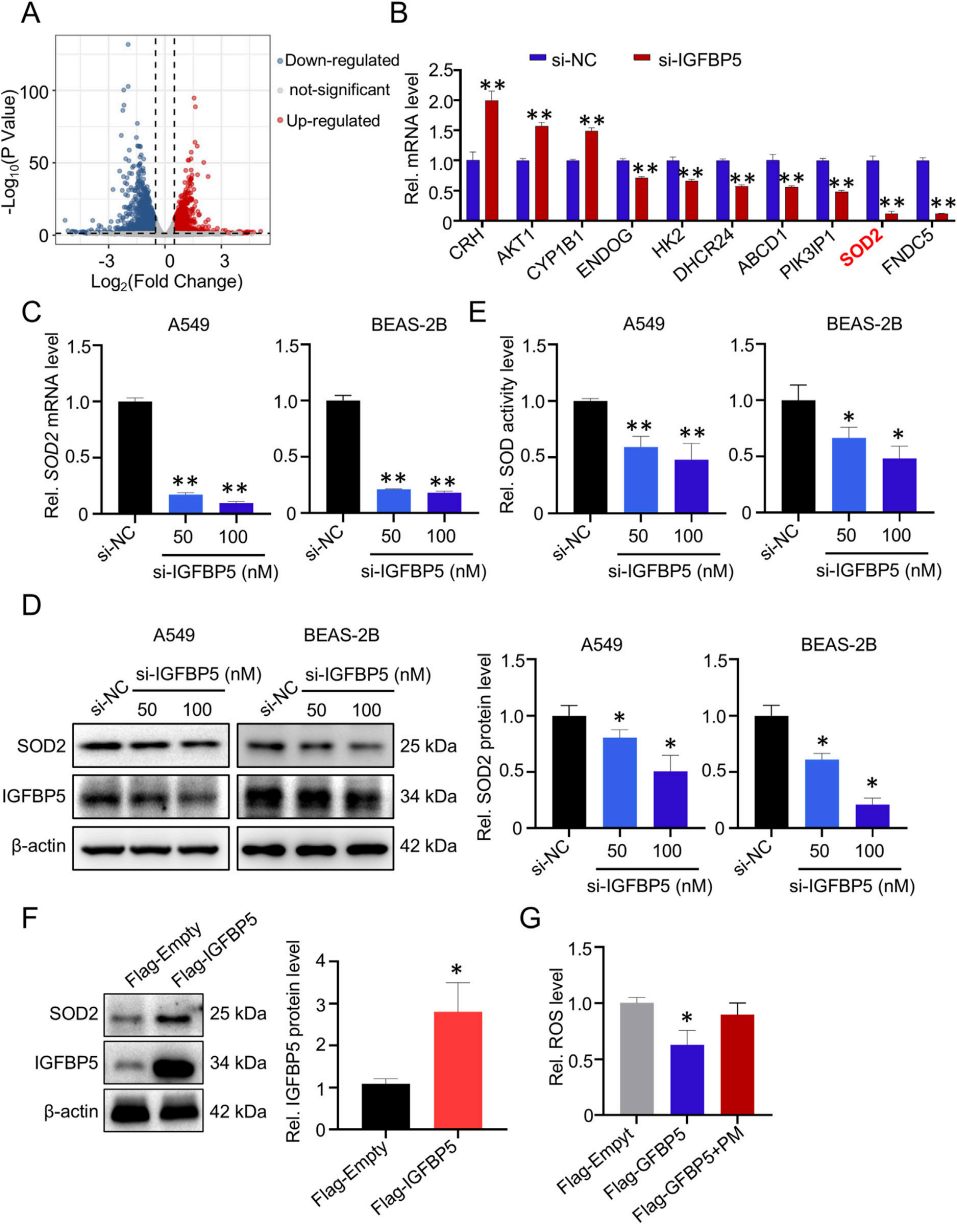
The expression of SOD2 is affected by IGFBP5. **(A)** Volcano plot of 793 dramatically deregulated (fold change >2) mRNAs in A549 cells transfected with si-IGFBP5. **(B)** Validation of the top ten deregulated mRNAs in si-IGFBP5 transfected cells via qRT-PCR. **(C, D)** The impact of IGFBP5 on the SOD2 level was determined through qRT-PCR **(C)** and Western blotting **(D)**. **(E)** Measurement of intracellular total SOD levels. **(F)** SOD2 levels was upregulated in cell transfected with IGFBP5 overexpression vector. **(G)** Overexpression of IGFBP5 inhibited the PM-induced ROS levels. Data are presented as mean ± SD (n = 3), *P < 0.05 and **P < 0.01 as indicated.

To gain a deeper understanding of the mechanism by which IGFBP5-mediated oxidative damage in the lungs, RNA-sequencing was performed after knockdown of IGFBP5 by siRNA in A549 cells. As illustrated in Figure 3A, si-IGFBP5 treatment was found to have a considerable impact on the intracellular transcriptome; and a total of 793 deregulated (fold change >2) mRNAs were identified in the si-IGFBP5 (100 nM) group, compared to the si-NC group. Subsequently, qRT-PCR was conducted to evaluate the expression levels of the top ten differentially expressed mRNAs in cells treated with si-IGFBP5 (100 nM) (Figure 3B). Of those, we observed that the mRNA level of SOD2, a key enzyme responsible for maintaining cellular ROS homeostasis, was significantly decreased by si-IGFBP5 (decrease by 88.1%). These results prompted us to ask whether IGFBP5 participated in the regulation of cellular oxidative damage by affecting SOD2. To this end, we measured SOD2 mRNA and protein levels in cells treated with different concentrations of si-IGFBP5. As illustrated in Figures 3C,D, si-IGFBP5 decreased SOD2 mRNA and protein levels in a dose-dependent manner. Next, the biochemical analysis demonstrated a considerable decrease in SOD enzyme activity upon administration of si-IGFBP5 (100 nM) in both A549 and BEAS-2B cells (A549 decrease by 53%; BEAS-2B decrease by 48.3%) (Figure 3E). To further verify our results, we overexpressed IGFBP5 in A549 cells and found that IGFBP5 overexpression upregulated SOD2 protein levels (Figure 3F). Consistently, IGFBP5 overexpression inhibited the ability of PM exposure to induce elevated ROS levels (Figure 3G). Collectively, our findings indicate that IGFBP5 silencing could inhibit SOD2 and promote ROS production in lung cells.

### 3.3 ERK1/2 signaling pathway is a requisite step for IGFBP5 to regulate SOD2

**FIGURE 4:**
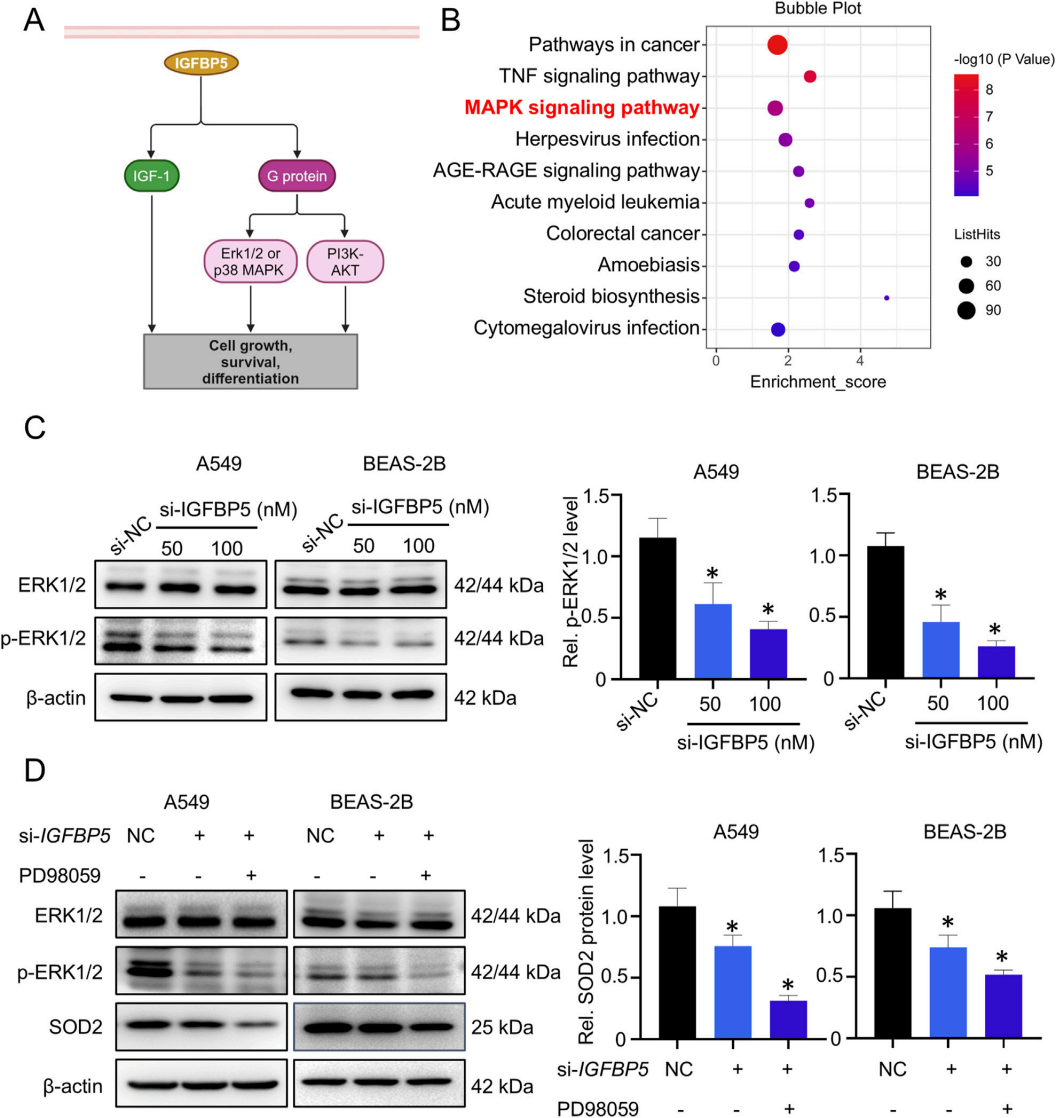
ERK1/2 pathway is involved in IGFBP5-mediated downregulation of SOD2 expression. **(A)** A model diagram illustrating the molecular interactions of IGFBP5 in cells. **(B)** Functional analysis of differentially expressed genes was conducted using KEGG. **(C)** Western blotting analysis was used to detect the ERK1/2 and p-ERK1/2 levels in cells transfected with or without si-IGFBP5. **(D)** The regulatory capacity of IGFBP5 on SOD2 protein level in PD98059-treated cells using Western blotting. Data are presented as mean ± SD (n = 3), *P < 0.05 and **P < 0.01 as indicated.

IGFBP5 is known to exert its effects via both IGF-I dependent and IGF-I independent pathways (Figure 4A). To explore how IGFBP5 affects SOD2 expression, KEGG enrichment analysis was employed to explore the functions of differentially expressed genes in IGFBP5 knockdown cells. Unexpectedly, the classical IGF-I dependent signaling pathway was not enriched, whereas non-classical signaling pathway, such as MAPK (Güllü et al., 2012), was markedly enriched (Figure 4B). To validate this result, we first extracted total RNA from the si-IGFBP5 treated cells and used qRT-PCR to evaluate the effects of IGFBP5 on downstream target genes of the IGF-I signaling pathway. As shown in Supplementary Figure S3, although IGFBP5 was absent, the expressions of IRS2 and SHP2 remained unchanged in A549 and BEAS-2B cells, indicating that IGFBP5 did not affect the expression of SOD2 through the IGF-I dependent signaling pathway. We then measured the levels of MAPK pathway levels, which consisted of three distinct components: ERK1/2, JNK and p38. As illustrated in Figure 4C and Supplementary Figure S4, si-IGFBP5 only reduced the phosphorylation level of ERK1/2 (p-ERK1/2) (A549: decrease by 33.6% and 51.3% at 50 μg/mL and 100 μg/mL, respectively; BEAS-2B: decrease by 46.8% and 64.1% at 50 μg/mL and 100 μg/mL, respectively), suggesting that ERK1/2 pathway was disrupted by IGFBP5. To further explore whether IGFBP5 affected SOD2 expression through the ERK1/2 pathway, the ERK1/2 pathway inhibitor PD98059 was used. Results showed that expressions of p-ERK1/2 and SOD2 in cells were inhibited by si-IGFBP5 (SOD2 decreased by 24.1% in A549 cells and 26.2% in BEAS-2B cells) (Figure 4D). Furthermore, the incorporation of PD98059 resulted in a more decrease in both p-ERK1/2 and SOD2 levels (SOD2 decreased by 68.5% in A549 cells and 48.3% in BEAS-2B cells) (Figure 4D). These findings suggest that ERK1/2 pathway plays a crucial role in the regulation of SOD2 by IGFBP5.

### 3.4 MiR-33a-5p targets IGFBP5 transcript

**FIGURE 5:**
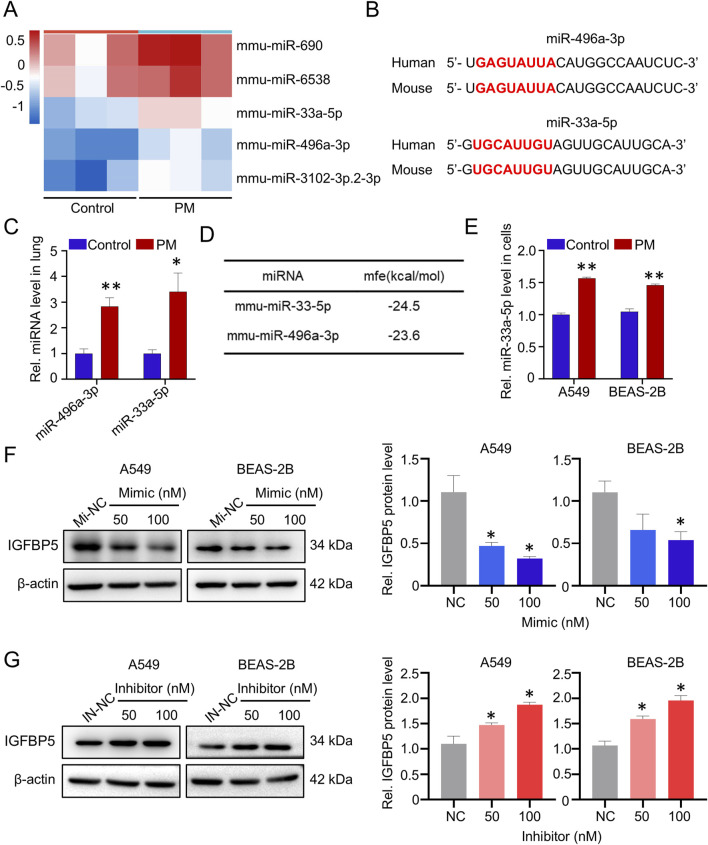
PM exposure upregulates miR-33a-5p, and miR-33a-5p negatively correlates with IGFBP5. **(A)** Heatmap and volcano plot of 5 dramatically deregulated miRNA (fold change >2) in lung tissue (n = 3). **(B)** Sequence alignment analysis of miR-33a-3p in humans and mouse. Red text highlighted the miRNAs seed region. **(C)** Effect of PM on miRNAs in lung tissue. **(D)** miRNAs were predicted to target IGFBP5 by using RNAhybrid tools. **(E)** Validation of the miR-33a-5p level under PM exposure via qRT-PCR. **(F, G)** Western blotting analysis was used to detect the IGFBP5 protein expression in cells transfected with miR-33a-5p mimic **(F)** or inhibitor **(G)**. Data are presented as mean ± SD (n = 3), *P < 0.05 and **P < 0.01 as indicated.

We next wanted to elucidate the mechanism responsible for PM-induced dysregulation of IGFBP5. As illustrated in Figure 5A, compared with control group, 5 deregulated (fold-change >2) miRNAs were identified in the PM exposure group. Further sequence comparison suggested that only miR-496a-3p and miR-33a-5p were highly conserved in human and mice (Figure 5B), and both of them were significantly upregulated in mice exposed to PM, as compared to the control group (Figure 5C). Next, the miRNAs that potentially regulate IGFBP5 were predicted by systematic *in silico* analysis. Results derived from RNAhybrid tools (bibiserv.cebitec.uni-bielefeld.de/rnahybrid/) showed that both miR-496a-3p and miR-33a-5p were predicted to target the 3′UTR region of IGFBP5, but miR-33a-5p exhibited the lowest binding free energy with IGFBP5, implying that miR-33a-5p was a strong contender for regulating IGFBP5 (Figure 5D). To test this idea, we first measured the expression of miR-33a-5p in cells, and found that the expression level of miR-33a-5p in the PM-exposed group was higher than that in the control group (Figure 5E). We then synthesized miRNA mimic and inhibitor, respectively, and determined their efficiencies by qRT-PCR (Supplementary Figures S5A, B). As expected, the overexpression of miR-33a-5p led to a substantial reduction in the level of IGFBP5 protein, while the inhibition of miRNA had the opposite effect (Figures 5F,G), indicating that miR-33a-5p involved in regulation of IGFBP5 expression.

**FIGURE 6:**
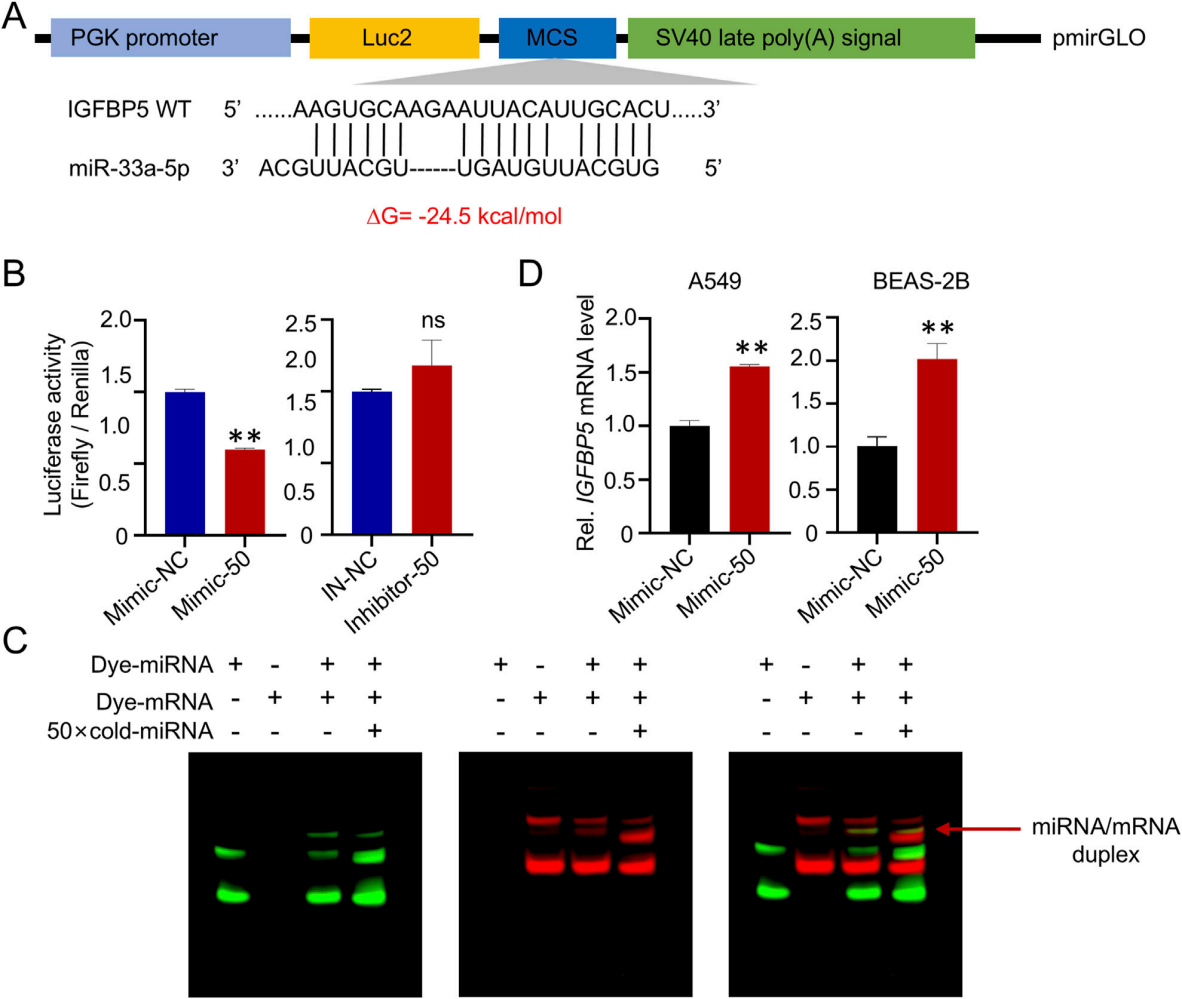
MiR-33a-5p suppresses the expression of IGFBP5 by binding directly to IGFBP5 mRNA. **(A)** The schematic diagram of reporter gene plasmid. **(B)** Dual-luciferase reporter gene analysis was conducted after 48 h co-transfection of plasmids (IGFBP5-WT) and miR-33a-5p mimic or miR-33a-5p-inhibitor in HEK 293 T cells. **(C)** FREMSA assay was employed to explore the interaction relationship between miR-33a-5p and IGFBP5 *in vitro*. **(D)** miR-33a-5p upregulates IGFBP5 mRNA levels. Data are presented as mean ± SD (n = 3), *P < 0.05 and **P < 0.01 as indicated.

To further explore the regulatory effect of miR-33a-5p on IGFBP5, we constructed a recombinant plasmid IGFBP5-3′UTR-WT (Figure 6A). As shown in Figure 6B, miR-33a-5p mimics, but not its inhibitor, significantly suppressed the luciferase activities produced by IGFBP5-3′UTR-WT in cells (decreased by 40.2%). Furthermore, *in vitro* FREMSA assays were adopted to evaluate the sequence specificity of the binding between miR-33a-5p and 3′UTR of IGFBP5 (Figure 6C). A notable shift in mobility was observed in lane 2 upon mixing the dye-labeled miR-33a-5p and IGFBP5 oligonucleotides, suggesting the formation of miRNA-mRNA complexes. While excess unlabeled miRNA oligonucleotides reduced the stable miRNA/mRNA complexes (lane 3), further indicating that miR-33a-5p could directly bind to the 3′UTR of IGFBP5 transcript in a sequence-specific manner. Generally, the outcome of pairing miRNA and its target mRNA is either transcript degradation or impaired transcript translation efficiency. As shown in Figure 6D, cells treated with miRNA mimic did not reduce the IGFBP5 mRNA level. Given that overexpression of miRNA has been found to suppress IGFBP5 protein level, our results indicate that miR-33a-5p could directly target the 3′UTR of IGFBP5, thereby reducing translation.

### 3.5 MiR-33a-5p inhibits IGFBP5 expression with the participation of AGO2

**FIGURE 7:**
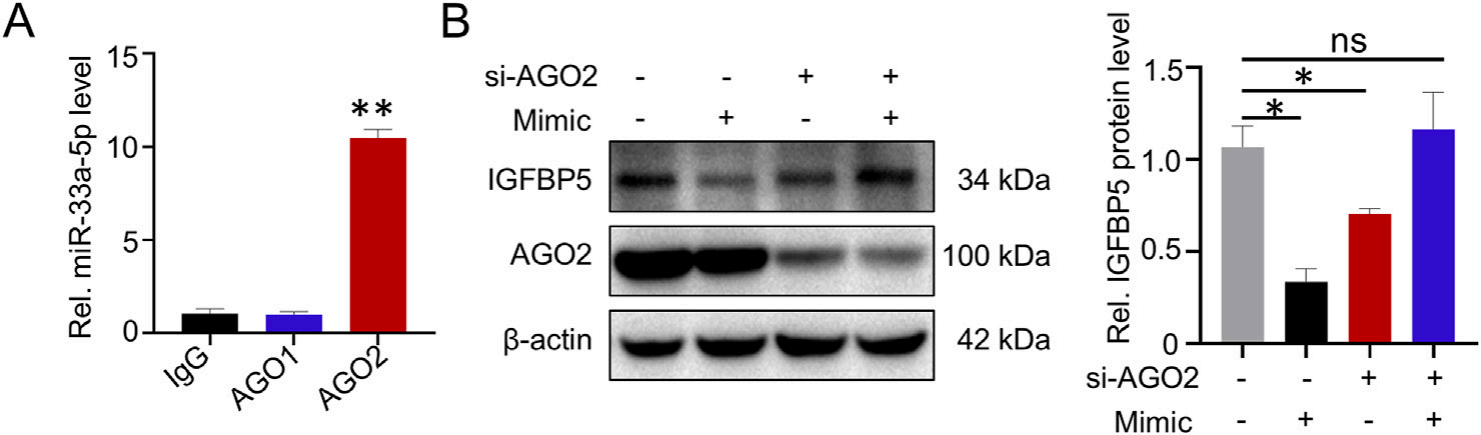
miR-33a-5p/AGO2 axis regulates IGFBP5 expression. **(A)** RIP assay was conducted to detect the interaction between miR-33a-5p and AGO proteins in A549. **(B)** The regulatory capacity of miR-33a-5p on IGFBP5 protein level in AGO2 knockdown cells. Data are presented as mean ± SD (n = 3), *P < 0.05 and **P < 0.01 as indicated.

It has been reported that miRNAs regulate target gene expression by loading AGO proteins, especially AGO1 and AGO2, we next wanted to explore whether the regulatory effect of miR-33a-5p on IGFBP5 required AGO protein. RIP assays results showed that miR-33a-5p could interact with AGO2, but not with AGO1 (AGO1: 1.12-fold enrichment; AGO2: 10.43-fold enrichment) (Figure 7A). To further investigate the role of AGO2 in miR-33a-5p function, siRNAs targeting the AGO2 gene and miR-33a-5p mimic were co-transfected into A549 cells. We observed that the inhibition of IGFBP5 protein levels induced by miR-33a-5p mimics was blocked after knocking down AGO2 protein (Supplementary Figure S6; Figure 7B), suggesting that AGO2 was involved in the regulation of IGFBP5 expression by miR-33a-5p.

### 3.6 MiR-33a-5p enhances cell oxidative damage by down-regulating IGFBP5

**FIGURE 8:**
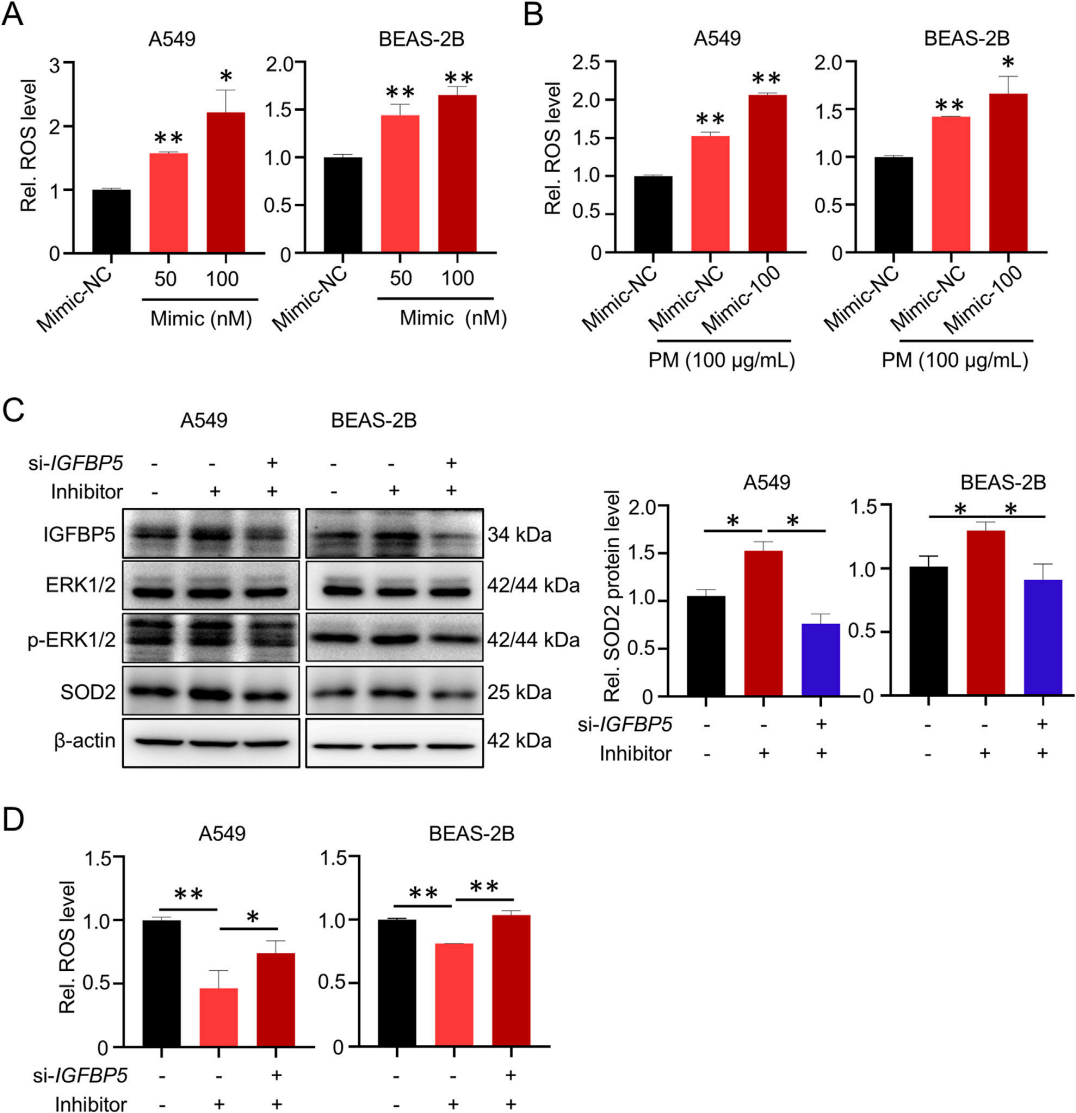
miR-33a-5p increases ROS levels through IGFBP5 in A549 and BEAS-2B cells. **(A)** miR-33a-5p increases ROS levels in cells. **(B)** The combination of miR-33a-5p and PM can synergistically upregulate cellular ROS levels. **(C)** Western blotting analysis detected the IGFBP5-ERK1/2-SOD2 pathway levels in miR-33a-5p inhibitor treated cells co-transfected with or without si-IGFBP5. **(D)** Measurement of intracellular ROS levels. Data are presented as mean ± SD (n = 3), *P < 0.05 and **P < 0.01 as indicated.

We next wanted to verify whether miR-33a-5p promoted cell oxidative damage by repressing IGFBP5 expression. As shown in Figure 8A, miR-33a-5p overexpression could significantly upregulate the intracellular ROS level in A549 and BEAS-2B cells. Intriguingly, the combination of the miR-33a-5p mimic and PM could further elevate cellular ROS levels (Figure 8B). Moreover, we transfected si-IGFBP5 into miR-33a-5p inhibitor-treated cells to suppress the expression of IGFBP5. Western blotting analysis showed that miRNA inhibitor increased IGFBP5, SOD2 and p-ERK1/2 levels, while the integration of miRNA inhibitor and si-IGFBP5 exhibited an inhibitory effect on these components (Figure 8C). Consistently, the inhibitory effect of miRNA inhibitors on ROS levels was alleviated following the introduction of si-IGFBP5 (Figure 8D). Taken together, these results indicate that miR-33a-5p is involved in the regulation of ROS levels by targeting IGFBP5.

## 4 Discussion

Our study aimed to investigate the association between IGFBP5 and PM-induced lung injury. We uncover several significant findings, including (1) IGFBP5 is involved in oxidative damage of lung tissue induced by PM exposure; (2) ERK1/2 signaling pathway, but not IGF-I signaling pathway, is a necessary step for IGFBP5 to regulate SOD2; (3) The upregulated miR-33a-5p inhibits IGFBP5 translation by forming a silencing complex with AGO2 protein; (4) Targeted inhibition of miR-33a-5p could alleviate PM-induced lung injury. These observations provide critical insights into the underlying mechanisms of real-ambient PM-induced lung injury.

As oxidative damage plays a key role in the pathogenesis of PM-induced lung injury, a plethora of antioxidant components have been developed with the aim of providing therapeutic benefit. For example, fucosterol is a natural plant ingredient isolated and purified from the brown alga Sargassum. There is evidence that fucosterol reduces ROS levels in lung epithelial cells by elevating a series of antioxidant enzymes such as SOD, glutathione (GSH) and catalase (CAT), thereby ameliorating PM-induced cell damage (Fernando et al., 2019). Additionally, Xin et al., found that vitamin D3 could alleviate PM-induced oxidative damage and inflammatory response by inhibiting the p38/NF-κB/NLRP3 signaling pathway (Xin et al., 2019). In agreement with previous studies, our results showed that elevated level of ROS was also a critical event in the pathogenesis of lung injury induced by real-ambient PM exposure. Therefore, elucidating the fundamental molecular processes underlying PM-induced oxidative damage responses in lung tissue will be of significant value in developing effective therapeutic strategies to combat PM-induced exacerbations of lung disease.

IGFBP5 was initially reported to be associated with tumors. For example, Taylor et al. reported that IGFBP5 could predict prognosis of primary breast tumors in a dynamic manner (Taylor et al., 2010). Additionally, low serum IGFBP5 levels have been proven to correlate strongly with a positive nodal status, which provides diagnostic value for identifying non-small cell lung cancer progression and patient outcome (Shersher et al., 2011). However, along with intensive research, the scientific community has gained a deeper insight into the function of IGFBP5, for example, IGFBP5 can sense extracellular stimuli and induce a series of cellular events. 2,3,7,8-tetrachlorodibenzo-p-dioxin (TCDD) is a persistent organic pollutant that has been widely distributed in the environment. Studies have shown that TCDD-induced toxicity in estrogen receptor-positive tissues is largely associated with the alteration in IGFBP5 expression (Tanaka et al., 2007). In this study, we found that IGFBP5 exhibited a significant change in response to PM exposure. Further studies showed that IGFBP5 was involved in the changes in the redox state of lung cells induced by PM, which undoubtedly expanded the function scope of IGFBP5, and suggested that IGFBP5 may be a good marker for PM-induced lung injury.

Traditionally, IGFBP5 has been considered to affect cell function mainly through the IGF-I signaling pathway. However, our KEGG analysis showed that the classic IGF-I dependent signaling pathway was not enriched in A549 cells with IGFBP5 knocked down. Meanwhile, knockdown of IGFBP5 did not disturb the expression of downstream genes in the IGF-I signaling pathway, suggesting that other pathways may be activated. Recently, PI3K/Akt and MAPK signaling pathway have been reported to be necessary for IGFBP5-enhanced cell growth, cell migration and fibrosis. In our research, we found that the MAPK, but not PI3K/Akt, was significantly enriched in the case of IGFBP5 knockdown. Notably, although MAPK pathway contains three classic components: ERK1/2, JNK and p38 (Rui et al., 2016; Song et al., 2020), our results showed that only ERK1/2 was significantly affected after the knockdown of IGFBP5.

In recent years, researchers have found that the epigenetic alteration, mainly including DNA methylation, histone acetylation, and noncoding RNA, may be an important interface between environmental pollutants and human health (Angrish et al., 2018). MiRNAs are a class of single-stranded noncoding RNAs with a length of approximately 22 nt. Extensive studies have shown that miRNA expression patterns can be altered in response to environmental cues. Meanwhile, miRNAs have been found to play important roles in fundamental processes, including proliferation, development, differentiation, inflammation, and apoptosis. These results undoubtedly stimulated interest in these tiny molecules. With this background, the hypothesis that PM-induced cytotoxicity might also be produced through changes in miRNA expression was proposed and subsequently validated. For example, Xu et al. reported that persistent airway inflammation caused by PM2.5 exposure was associated with miR-155-driven M1 macrophage polarization (Xu et al., 2024). Additionally, long-term PM2.5 exposure could promote fibroblast activation and excessive deposition of extracellular matrix (ECM) by interacting with the miRNA network, such as miR-21 (Dai et al., 2019), miR-150-5p (Lu et al., 2017) and miR-200c (Zhong et al., 2016), ultimately leading to irreversible pulmonary structural remodeling. Recent studies have shown that the increased risk of lung cancer caused by PM exposure is related to miRNA levels. For example, Qi et al. showed that miR-199a could suppress the process of lung cancer induced by PM2.5 exposure by inhibiting the production of inflammatory factors, epithelial-mesenchymal transition (EMT), and migration of lung cancer cells (Qi et al., 2021). Nonetheless, compared to the quantity identified through sequencing, only a handful of the exact mechanism of action in miRNA have been identified. In this study, we also found alterations in miRNA expression profiles in response to a real-ambient PM exposure, and identified a series of differentially expressed miRNAs. In particular, we found that upregulated miR-33a-5p could target and regulate IGFBP5. This result not only deepens the understanding of the function of IGFBP5, but also expands the molecular function of miR-33a-5p in PM-induced lung damage.

Results derived from *in silico* analysis and *in vitro* experiments showed that IGFBP5 was targeted and negatively regulated by miR-33a-5p, suggesting that inhibition of miR-33a-5p may be a viable antioxidant therapeutic option for preventing or treating a PM-induced lung injury. In fact, several studies have been conducted to elucidate the potential therapeutic mechanisms of miRNAs in PM-induced lung injury. For example, Li et al. showed that miR-486 mimic treatment could repress PM-induced ROS overproduction and improve the apoptosis by negatively regulating phosphatase and tensin homolog (PTEN)/Forkhead box O1 (FOXO1) in lung epithelial A549 cells (Li et al., 2018), suggesting that increasing the expression level of miR-486 contributed to alleviate PM-induced lung injury. Although the exact mechanism of miR-33a-5p needs to be further explored, our results provide a potential target for the treatment of PM-induced oxidative damage in lung tissue.

## 5 Conclusion

**FIGURE 9:**
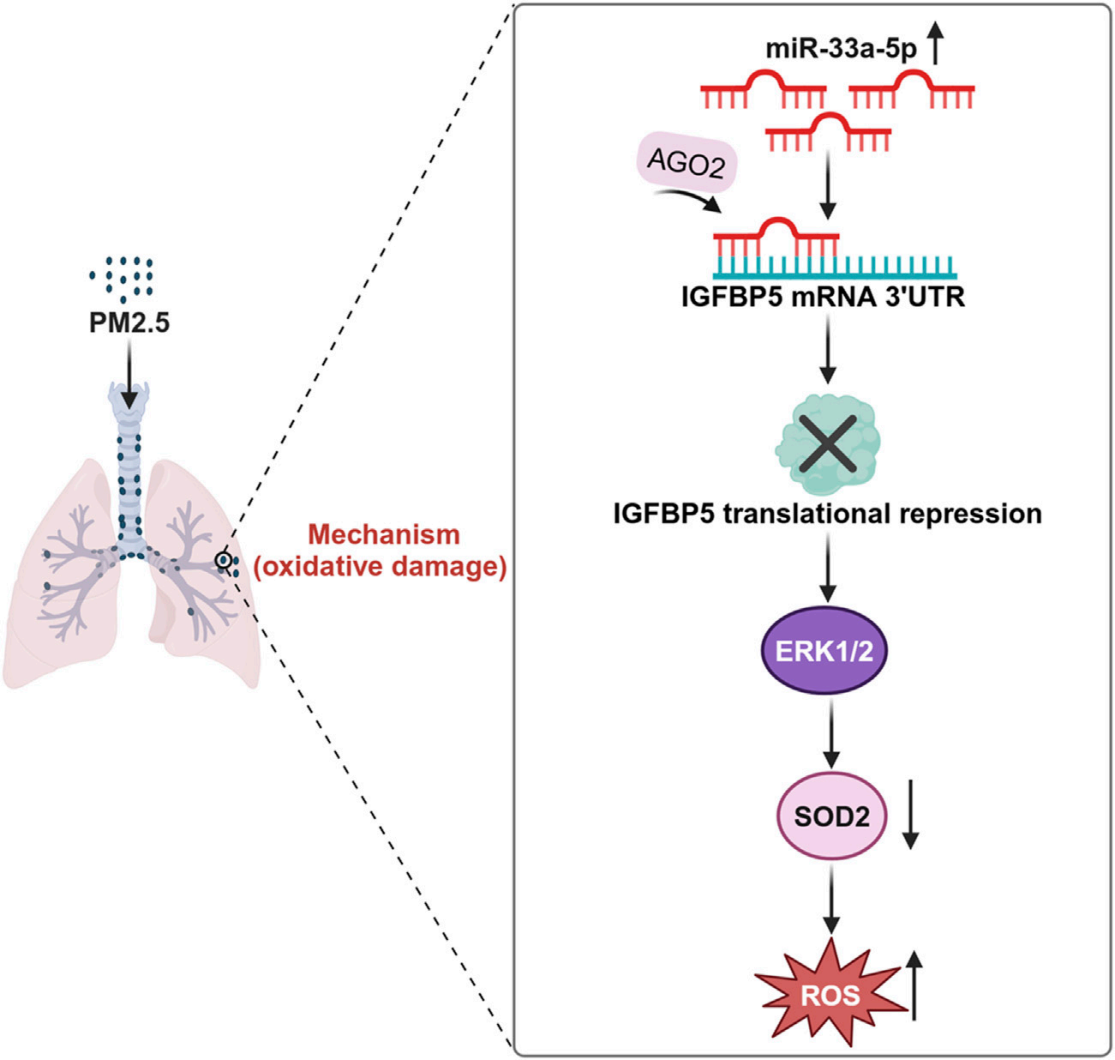
Schematic diagram of the mechanism by which IGFBP5 participates in PM exposure-induced lung injury.

In summary, we uncovered a novel mechanistic role of IGFBP5 in PM-induced lung injury. Our results suggested that the downregulation of IGFBP5 induced by PM exposure suppressed the ERK1/2-SOD2 pathway, thus exacerbating oxidative damage in lung cells. Furthermore, we systematically investigated the epigenetic regulation of IGFBP5 and revealed significant alterations in miRNA profile in PM-exposed lung tissues. Through comprehensive analysis, we identified miR-33a-5p as a key post-transcriptional regulator that directly targets the 3′-UTR of IGFBP5 mRNA to repress its translation (Figure 9). These mechanistic insights highlight IGFBP5 as both a critical pathophysiological mediator and a promising therapeutic target for mitigating real-ambient PM-induced oxidative damage.

## Data Availability

The original contributions presented in the study are publicly available. This data can be found here: https://www.ncbi.nlm.nih.gov/bioproject/PRJNA1279203.

## Abbreviations

PM, particulate matter; MDA, malondialdehyde; IGFBP5, insulin-like growth factor-binding protein 5; ROS, reactive oxygen species; SOD2, superoxide dismutase 2; 3′UTR, 3′untranslated region; MAPK, mitogen-activated protein kinase; ERK1/2, extracellular signal-related kinases 1 and 2.

## References

[B1] Allard J. B. Duan C. (2018). IGF-binding proteins: why do they exist and why are there so many? Front. Endocrinol. 9, 117. 10.3389/fendo.2018.00117 PMC5900387 29686648

[B2] Angrish M. M. Allard P. McCullough S. D. Druwe I. L. Helbling Chadwick L. Hines E. (2018). Epigenetic applications in adverse outcome pathways and environmental risk evaluation. Environ. health Perspect. 126, 045001. 10.1289/EHP2322 29669403 PMC6071815

[B3] Bach L. A. (2018). IGF-binding proteins. J. Mol. Endocrinol. 61, T11–t28. 10.1530/JME-17-0254 29255001

[B4] Chen L. Wu L. Cheng X. Huang J. Peng J. (2024). Effects of PM2.5 on mucus hypersecretion in airway through miR-133b-5p/EGFR/Claudin1/MUC5AC axis. Aging 16, 8472–8483. 10.18632/aging.205785 38809424 PMC11164504

[B5] Dai L. Chen F. Zheng Y. Zhang D. Qian B. Ji H. (2019). miR-21 regulates growth and EMT in lung cancer cells via PTEN/Akt/GSK3β signaling. Front. Biosci. Landmark Ed. 24, 1426–1439. 10.2741/4788 31136988

[B6] Deng Y. Yang X. Hua H. Zhang C. (2022). IGFBP5 is upregulated and associated with poor prognosis in colorectal cancer. Int. J. general Med. 15, 6485–6497. 10.2147/IJGM.S370576 PMC9365118 35966504

[B7] Dittmer J. (2022). Biological effects and regulation of IGFBP5 in breast cancer. Front. Endocrinol. 13, 983793. 10.3389/fendo.2022.983793 PMC9453429 36093095

[B8] Fan T. Y. Xu L. L. Zhang H. F. Peng J. Liu D. Zou W. D. (2024). Comprehensive analyses and experiments confirmed IGFBP5 as a prognostic predictor based on an aging-genomic landscape analysis of ovarian cancer. Curr. cancer drug targets 24, 760–778. 10.2174/0115680096276852231113111412 38018207

[B9] Fernando I. P. S. Jayawardena T. U. Kim H. S. Lee W. W. Vaas A. De Silva H. I. C. (2019). Beijing urban particulate matter-induced injury and inflammation in human lung epithelial cells and the protective effects of fucosterol from Sargassum binderi (Sonder ex J. Agardh). Environ. Res. 172, 150–158. 10.1016/j.envres.2019.02.016 30782534

[B10] Fuller R. Landrigan P. J. Balakrishnan K. Bathan G. Bose-O'Reilly S. Brauer M. (2022). Pollution and health: a progress update. Lancet Planet. health 6, e535–e547. 10.1016/S2542-5196(22)00090-0 35594895 PMC11995256

[B11] Gebert L. F. R. MacRae I. J. (2019). Regulation of microRNA function in animals. Nat. Rev. Mol. Cell Biol. 20, 21–37. 10.1038/s41580-018-0045-7 30108335 PMC6546304

[B12] Goobie G. C. Saha P. K. Carlsten C. Gibson K. F. Johannson K. A. Kass D. J. (2024). Ambient ultrafine particulate matter and clinical outcomes in fibrotic interstitial lung disease. Am. J. Respir. Crit. care Med. 209, 1082–1090. 10.1164/rccm.202307-1275OC 38019094 PMC11092946

[B13] Güllü G. Karabulut S. Akkiprik M. (2012). Functional roles and clinical values of insulin-like growth factor-binding protein-5 in different types of cancers. Chin. J. cancer 31, 266–280. 10.5732/cjc.011.10405 22313597 PMC3777492

[B14] Hou T. Liao J. Zhang C. Sun C. Li X. Wang G. (2018). Elevated expression of miR-146, miR-139 and miR-340 involved in regulating Th1/Th2 balance with acute exposure of fine particulate matter in mice. Int. Immunopharmacol. 54, 68–77. 10.1016/j.intimp.2017.10.003 29107863

[B15] Hou T. Zhu L. Wang Y. Peng L. (2024). Oxidative stress is the pivot for PM2.5-induced lung injury. Food Chem. Toxicol. Int. J. Publ. Br. Industrial Biol. Res. Assoc. 184, 114362. 10.1016/j.fct.2023.114362 38101601

[B16] Hu J. Yang L. Kang N. Wang N. Shen L. Zhang X. (2025). Associations between long-term exposure to fine particulate matter and its constituents with lung cancer incidence: evidence from a prospective cohort study in Beijing, China. Environ. Pollut. (Barking, Essex 1987) 368, 125686. 10.1016/j.envpol.2025.125686 39842494

[B17] Jiang S. Tong X. Yu K. Yin P. Shi S. Meng X. (2024). Ambient particulate matter and chronic obstructive pulmonary disease mortality: a nationwide, individual-level, case-crossover study in China. EBioMedicine 107, 105270. 10.1016/j.ebiom.2024.105270 39137570 PMC11367568

[B18] Li D. Zhang R. Cui L. Chu C. Zhang H. Sun H. (2019). Multiple organ injury in male C57BL/6J mice exposed to ambient particulate matter in a real-ambient PM exposure system in Shijiazhuang, China. Environ. Pollut. (Barking, Essex 1987) 248, 874–887. 10.1016/j.envpol.2019.02.097 30856503

[B19] Li J. Zhou Q. Liang Y. Pan W. Bei Y. Zhang Y. (2018). miR-486 inhibits PM2.5-induced apoptosis and oxidative stress in human lung alveolar epithelial A549 cells. Ann. Transl. Med. 6, 209. 10.21037/atm.2018.06.09 30023372 PMC6035978

[B20] Li X. Noell G. Tabib T. Gregory A. D. Trejo Bittar H. E. Vats R. (2021). Single cell RNA sequencing identifies IGFBP5 and QKI as ciliated epithelial cell genes associated with severe COPD. Respir. Res. 22, 100. 10.1186/s12931-021-01675-2 33823868 PMC8022543

[B21] Lu W. Zhang H. Niu Y. Wu Y. Sun W. Li H. (2017). Long non-coding RNA linc00673 regulated non-small cell lung cancer proliferation, migration, invasion and epithelial mesenchymal transition by sponging miR-150-5p. Mol. Cancer 16, 118. 10.1186/s12943-017-0685-9 28697764 PMC5504775

[B22] Qi H. Liu Y. Wang N. Xiao C. (2021). Lentinan attenuated the PM2.5 exposure-induced inflammatory response, epithelial-mesenchymal transition and migration by inhibiting the PVT1/miR-199a-5p/caveolin1 pathway in lung cancer. DNA cell Biol. 40, 683–693. 10.1089/dna.2020.6338 33902331

[B23] Ran S. Zhang J. Tian F. Qian Z. M. Wei S. Wang Y. (2025). Association of metabolic signatures of air pollution with MASLD: observational and Mendelian randomization study. J. Hepatol. 82, 560–570. 10.1016/j.jhep.2024.09.033 39349253

[B24] Rui W. Guan L. Zhang F. Zhang W. Ding W. (2016). PM2.5-induced oxidative stress increases adhesion molecules expression in human endothelial cells through the ERK/AKT/NF-κB-dependent pathway. J. Appl. Toxicol. JAT 36, 48–59. 10.1002/jat.3143 25876056

[B25] Shersher D. D. Vercillo M. S. Fhied C. Basu S. Rouhi O. Mahon B. (2011). Biomarkers of the insulin-like growth factor pathway predict progression and outcome in lung cancer. Ann. Thorac. Surg. 92, 1805–1811. 10.1016/j.athoracsur.2011.06.058 21945224

[B26] Song C. Wang S. Fu Z. Chi K. Geng X. Liu C. (2022). IGFBP5 promotes diabetic kidney disease progression by enhancing PFKFB3-mediated endothelial glycolysis. Cell Death Dis. 13, 340. 10.1038/s41419-022-04803-y 35418167 PMC9007962

[B27] Song L. Jiang S. Pan K. Du X. Zeng X. Zhang J. (2020). AMPK activation ameliorates fine particulate matter-induced hepatic injury. Environ. Sci. Pollut. Res. Int. 27, 21311–21319. 10.1007/s11356-020-08624-4 32270451

[B28] Tanaka J. Yonemoto J. Zaha H. Kiyama R. Sone H. (2007). Estrogen-responsive genes newly found to be modified by TCDD exposure in human cell lines and mouse systems. Mol. Cell. Endocrinol. 272, 38–49. 10.1016/j.mce.2007.04.008 17555868

[B29] Taylor K. J. Sims A. H. Liang L. Faratian D. Muir M. Walker G. (2010). Dynamic changes in gene expression *in vivo* predict prognosis of tamoxifen-treated patients with breast cancer. Breast cancer Res. BCR 12, R39. 10.1186/bcr2593 20569502 PMC2917034

[B30] Vaccarella E. Massimi L. Canepari S. (2025). Assessment of oxidative stress induced by atmospheric particulate matter: from acellular and cellular assays to the use of model and experimental organisms. Sci. total Environ. 965, 178651. 10.1016/j.scitotenv.2025.178651 39892228

[B31] Wang Y. Zhong Y. Zhang C. Liao J. Wang G. (2020). PM2.5 downregulates MicroRNA-139-5p and induces EMT in bronchiolar epithelium cells by targeting Notch1. J. Cancer 11, 5758–5767. 10.7150/jca.46976 32913469 PMC7477455

[B32] Xin L. Che B. Zhai B. Luo Q. Zhang C. Wang J. (2019). 1,25-Dihydroxy vitamin D(3) attenuates the oxidative stress-mediated inflammation induced by PM(2.5)via the p38/NF-κB/NLRP3 pathway. Inflammation 42, 702–713. 10.1007/s10753-018-0928-y 30430362

[B33] Xu H. Li X. Liu K. Huang P. Liu X. J. (2024). PM2.5 promotes macrophage-mediated inflammatory response through airway epithelial cell-derived exosomal miR-155-5p. J. Inflamm. Res. 17, 8555–8567. 10.2147/JIR.S482509 39539727 PMC11559224

[B34] Yasuoka H. Garrett S. M. Nguyen X. X. Artlett C. M. Feghali-Bostwick C. A. (2019). NADPH oxidase-mediated induction of reactive oxygen species and extracellular matrix deposition by insulin-like growth factor binding protein-5. Am. J. physiology Lung Cell. Mol. physiology 316, L644–l655. 10.1152/ajplung.00106.2018 PMC6483014 30810066

[B35] Yasuoka H. Yamaguchi Y. Feghali-Bostwick C. A. (2009). The pro-fibrotic factor IGFBP-5 induces lung fibroblast and mononuclear cell migration. Am. J. Respir. Cell Mol. Biol. 41, 179–188. 10.1165/rcmb.2008-0211OC 19131643 PMC2715907

[B36] Zhong X. Zheng L. Shen J. Zhang D. Xiong M. Zhang Y. (2016). Suppression of MicroRNA 200 family expression by oncogenic KRAS activation promotes cell survival and epithelial-mesenchymal transition in KRAS-driven cancer. Mol. Cell Biol. 36, 2742–2754. 10.1128/MCB.00079-16 27550813 PMC5064220

