# Supplementary material for: Role and mechanism of IGFBP5 in the real-ambient particulate matter exposure-induced chronic lung injury
Source: Front Pharmacol. 2025 Jun 25;16:1604301. doi: 10.3389/fphar.2025.1604301 (PMC12265953; doi:10.3389/fphar.2025.1604301)
Supplement: Supplementary file 1 [file Supplementaryfile1.docx]

**Supplementary information to:**

## Role and mechanism of IGFBP5 in the real-ambient particulate matter exposure-induced chronic lung injury

Ningning Chen^1, 2, 3^; Yuan Qi^3^, Wanli Ma^3^, Xiaoxiao Zhu^3^, Xiaoying Li^4, #^

^1^Department of Neonatology, Children's Hospital Affiliated to Shandong University (Jinan Children's Hospital), Jinan, China.

^2^School of Basic Medical Sciences, Shandong University, Jinan, China.

^3^School of Public Health, Qingdao University, Qingdao, China.

^4^Department of Neonatology, Children's Hospital Affiliated to Shandong University (Jinan Children's Hospital), Jinan, China.

**Content**

Figure S1 PM exposure induces oxidative damage in mouse lung tissue.

Figure S2 The effects of IGFBP5 on the inflammatory cytokine levels.

Figure S3 The effect of IGFBP5 on the IGF-I signaling pathways.

Figure S4 The effects of IGFBP5 on the JNK and P38 levels.

Figure S5 Verification of the efficiency of miR-33a-5p-related synthetic compounds.

Figure S6 Verification of the efficiency of siRNA-AGO2.

Supplementary Table 1 Sequences of primers used in this study.

Supplementary Table 2 Antibodies used in this study.

**
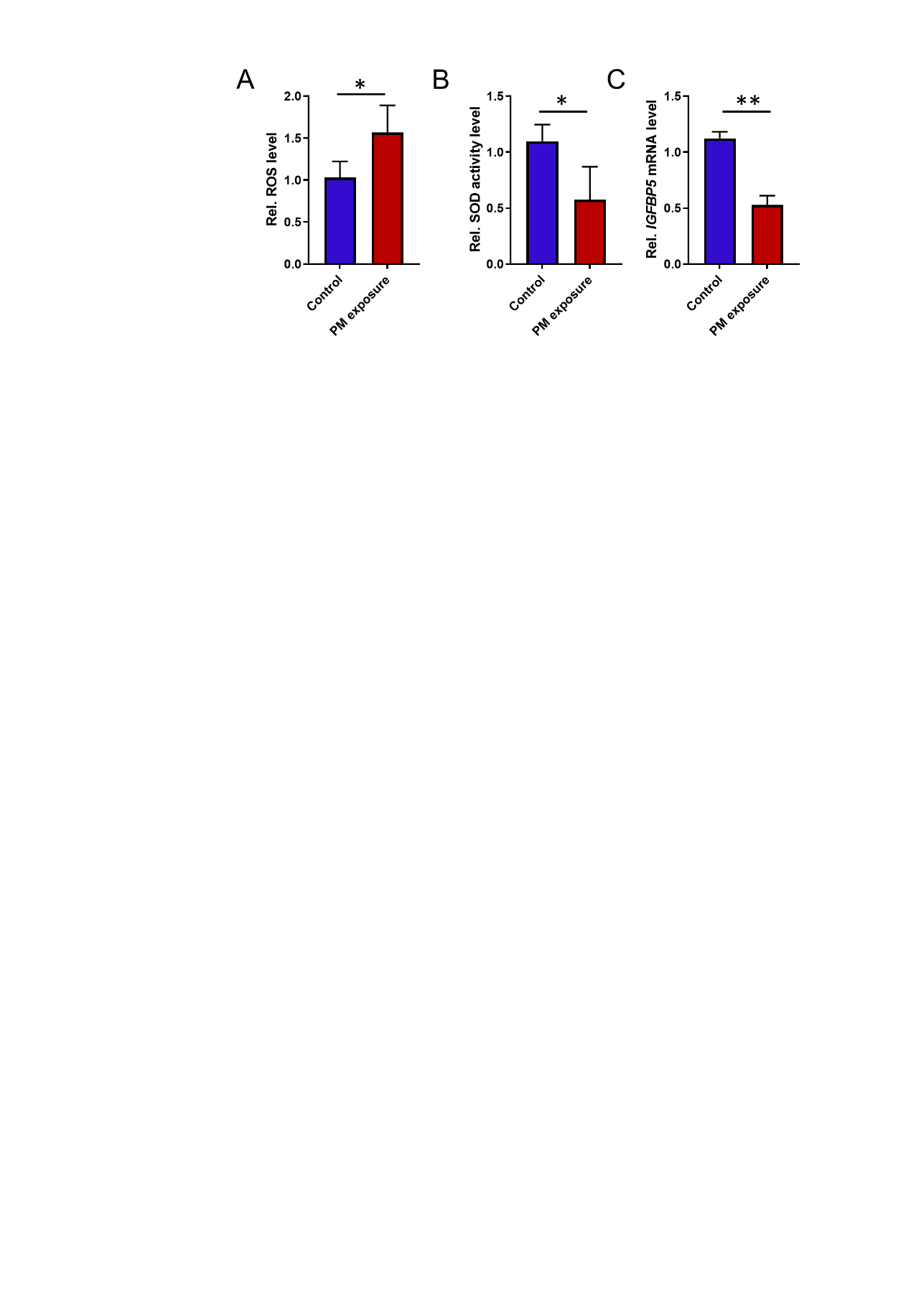
**

**Figure S1 PM exposure induces oxidative damage in mouse lung tissue.** **(A-B)** The impacts of PM exposure on the ROS **(A)** and SOD **(B)** levels. **(C)** The impacts of PM on the IGFBP5 level were determined through qRT-PCR. Data are presented as mean ± SD (n = 10), **P* < 0.05 and ***P* < 0.01 as indicated.

**
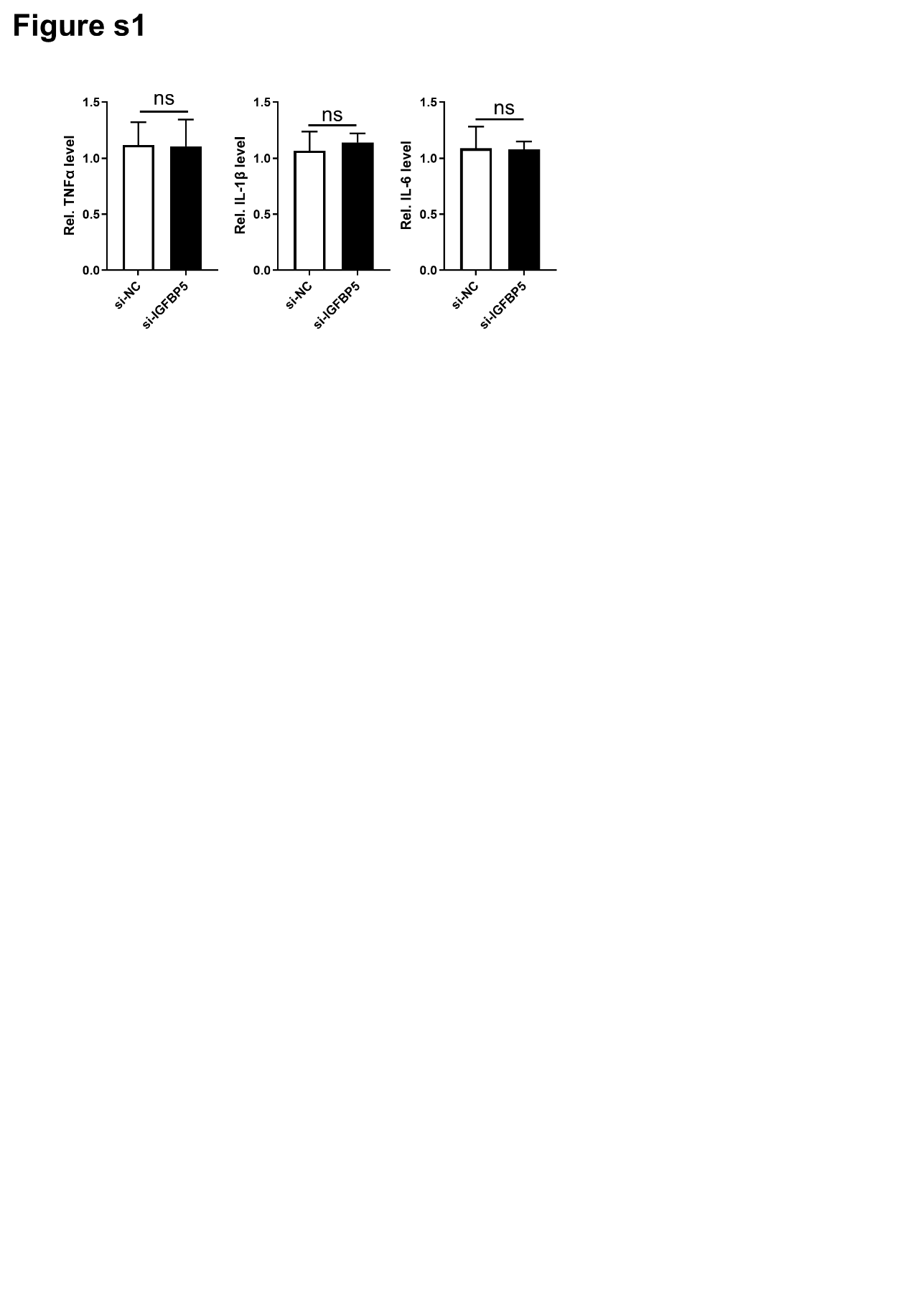
**

**Figure S2 The effects of IGFBP5 on the inflammatory cytokine levels.** The impacts of IGFBP5 on the TNFα, IL-1β and IL-6 level were determined through qRT-PCR. Data are presented as mean ± SD (n = 3), **P* < 0.05 and ***P* < 0.01 as indicated.

**
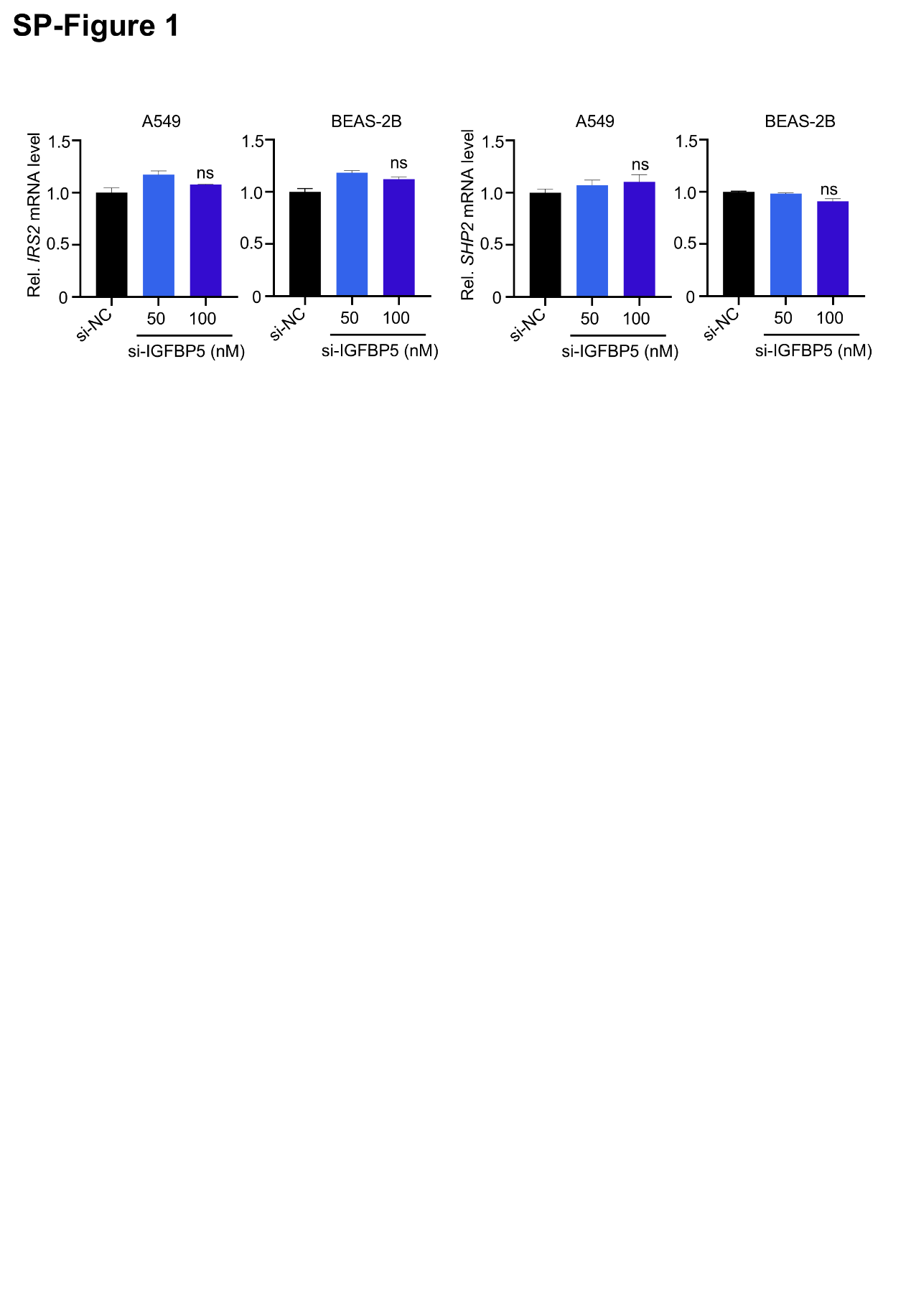
**

**Figure S3 The effect of IGFBP5 on the IGF-I signaling pathways.** The impact of IGFBP5 on the IPS2 and SHP2 level that are the downstream of IGF-I signaling pathways was determined through qRT-PCR. Data are presented as mean ± SD (n = 3), **P* < 0.05 and ***P* < 0.01 as indicated.


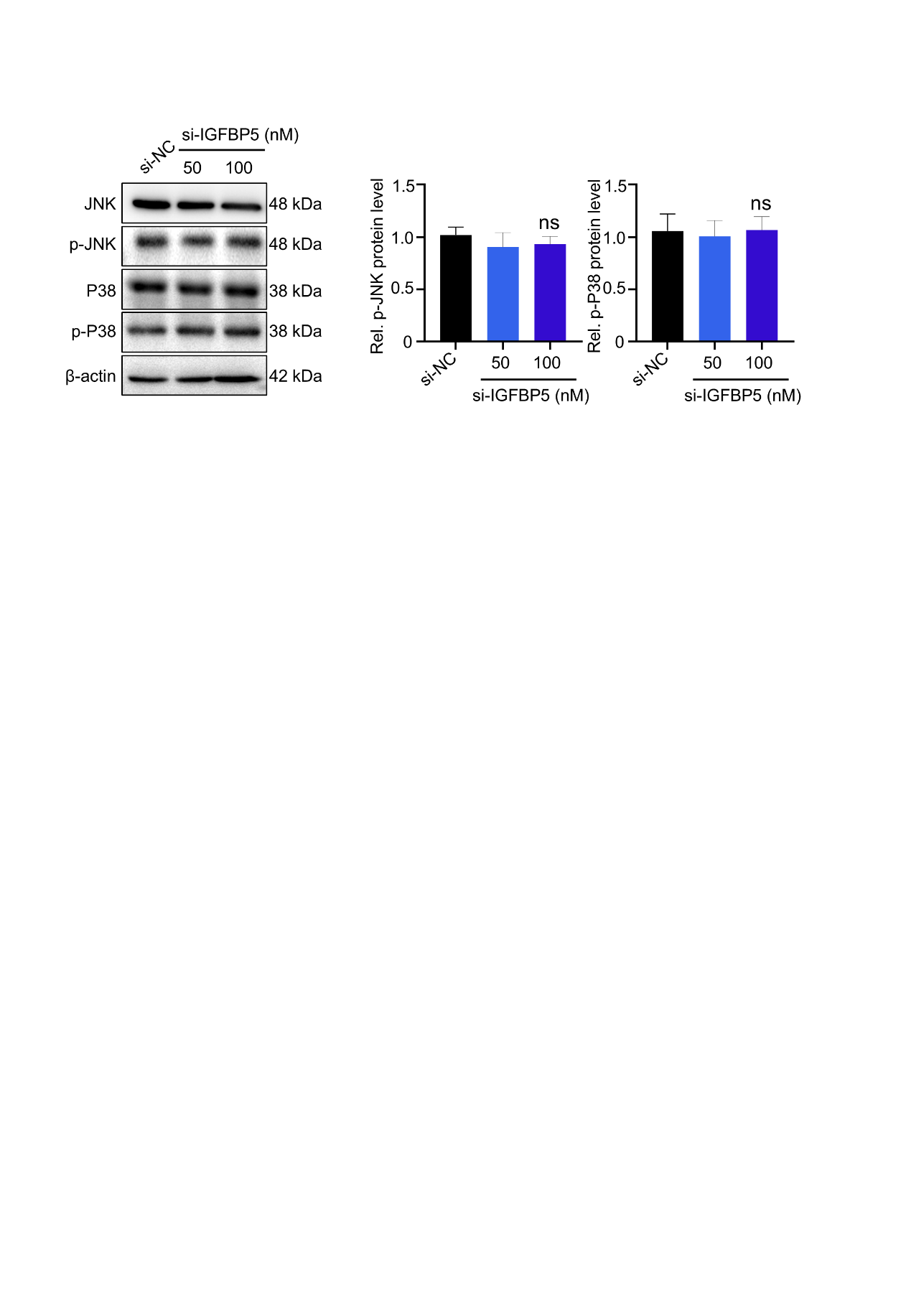


**Figure S4** **The effects of IGFBP5 on the JNK and P38 levels.** The impacts of IGFBP5 on the JNK and P38 level were determined through western blot. Data are presented as mean ± SD (n = 3), **P* < 0.05 and ***P* < 0.01 as indicated.


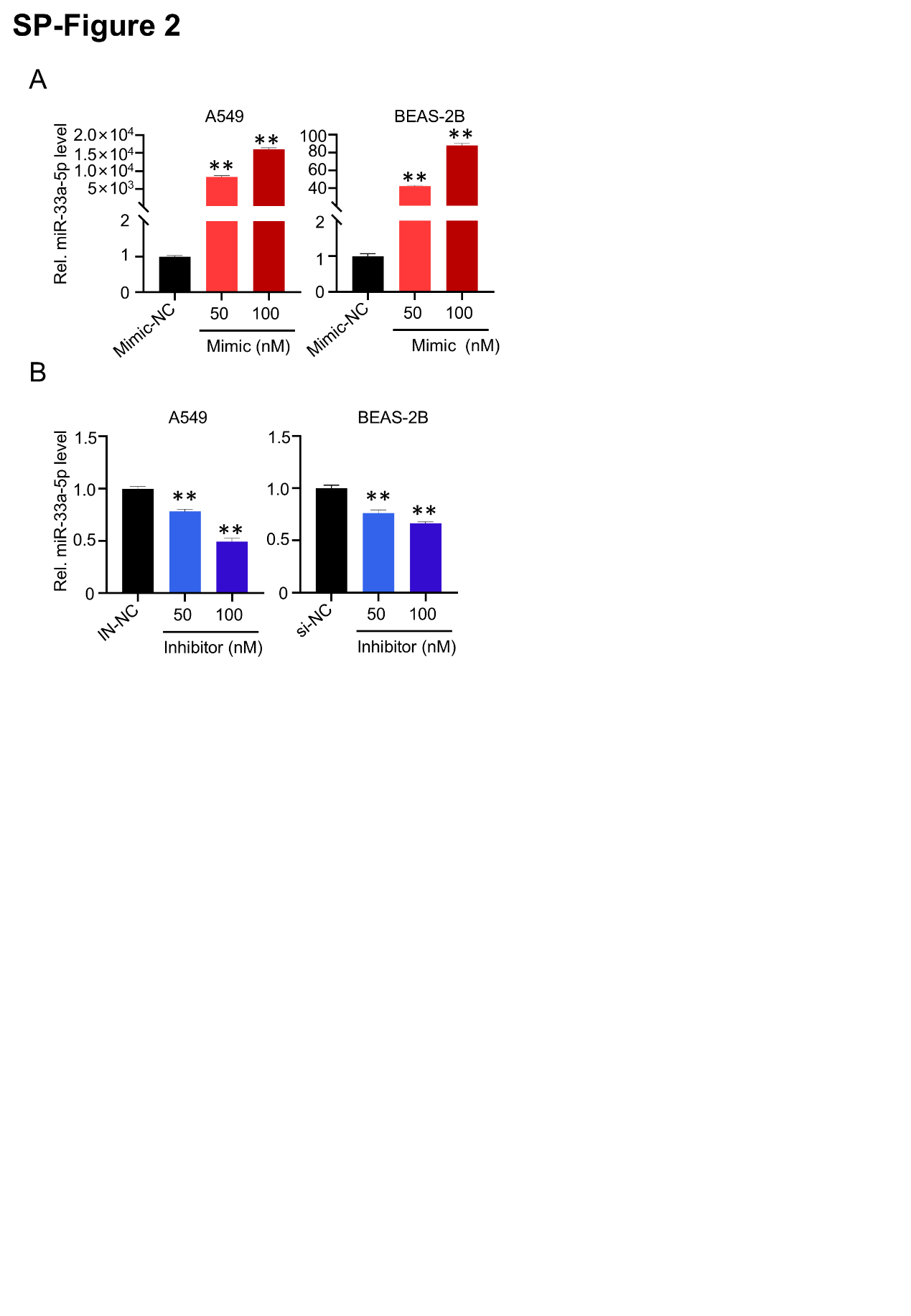


**Figure S5 Verification of the efficiency of miR-33a-5p-related synthetic compounds. (A-B)** Verification of the efficiency of miR-33a-5p mimic **(A)** and inhibitor **(B)** via qRT-PCR. Data are presented as mean ± SD (n = 3), **P* < 0.05 and ***P* < 0.01 as indicated.


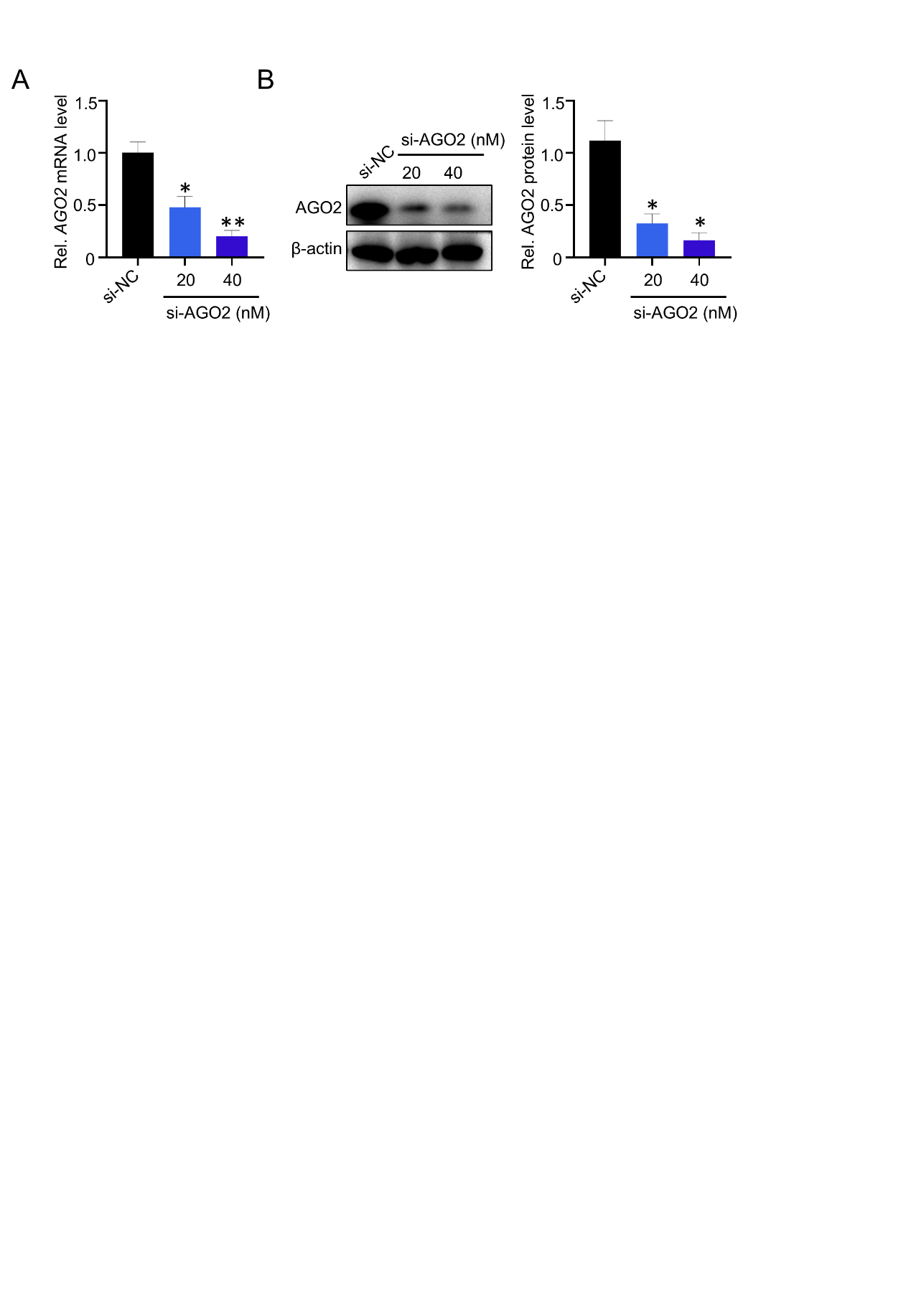


**Figure S4 Verification of the efficiency of siRNA-AGO2. (A-B)** Verification of the efficiency of siRNA-AGO2 via qRT-PCR (A) and western blot (B). Data are presented as mean ± SD (n = 3), **P* < 0.05 and ***P* < 0.01 as indicated.

**Supplementary Table 1** **Sequences of primers used in this study**

| Name | Sequence (5′–3′) |
| --- | --- |
| IGFBP5 | F: CGGGGTTTGCCTCAACGAA |
|  | R: TCTTGGGGGAGTAGGTCTCCT |
| SOD2 | F: CTGGACAAACCTCAGCCCTAAC |
|  | R: AACCTGAGCCTTGGACACCAAC |
| IRS2 | F: CCTGCCCCCTGCCAACACCT |
|  | R: TGTGACATCCTGGTGATAAAGCC |
| SHP2 | F: GACTTTTGGCGGATGGTGTTCC |
|  | R: CGGCGCTTTCTTTGACGTTCCT |
| TNFα | F: CTCTTCTGCCTGCTGCACTTTG |
|  | R: ATGGGCTACAGGCTTGTCACTC |
| IL-1β | F: CCACAGACCTTCCAGGAGAATG |
|  | R: GTGCAGTTCAGTGATCGTACAGG |
| IL-6 | F: AGACAGCCACTCACCTCTTCAG |
|  | R: TTCTGCCAGTGCCTCTTTGCTG |
| ACTB | F: CACCATTGGCAATGAGCGGTTC |
|  | R: CACCATTGGCAATGAGCGGTTC |
| miR-33a-5p | F: GGGGTGCATTGTAGTTGCA |
|  | R: CAGTGCGTGTCGTGGAGT |
| U6 | F: CTCGCTTCGGCAGCACA |
|  | R: AACGCTTCACGAATTTGCGT |

**Supplementary Table 2 Antibodies used in this study**

| Antibodies | Source | Identifier |
| --- | --- | --- |
| IGFBP5 | Abcam | ab254324 |
| SOD2 | Boster biological | BA4566 |
| ERK | Boster biological | BM4326 |
| p-ERK | Boster biological | BM4156 |
| β-actin | Boster biological | BM0627 |
